# Central venous catheter–related infections in hematology and oncology: 2020 updated guidelines on diagnosis, management, and prevention by the Infectious Diseases Working Party (AGIHO) of the German Society of Hematology and Medical Oncology (DGHO)

**DOI:** 10.1007/s00277-020-04286-x

**Published:** 2020-09-30

**Authors:** Boris Böll, Enrico Schalk, Dieter Buchheidt, Justin Hasenkamp, Michael Kiehl, Til Ramon Kiderlen, Matthias Kochanek, Michael Koldehoff, Philippe Kostrewa, Annika Y. Claßen, Sibylle C. Mellinghoff, Bernd Metzner, Olaf Penack, Markus Ruhnke, Maria J. G. T. Vehreschild, Florian Weissinger, Hans-Heinrich Wolf, Meinolf Karthaus, Marcus Hentrich

**Affiliations:** 1 Department I of Internal Medicine, Hematology and Oncology, Intensive Care Medicine, Center for Integrated Oncology Aachen Bonn Cologne Duesseldorf, University of Cologne, Kerpener Strasse 62, 50937 Cologne, Germany; 2 Department of Hematology and Oncology, Otto-von-Guericke University Magdeburg, Medical Center, Magdeburg, Germany; 3 Department of Hematology and Oncology, Mannheim University Hospital, Heidelberg University, Mannheim, Germany; 4 Clinic for Hematology and Oncology, University Medicine Göttingen, Georg-August-University, Göttingen, Germany; 5 Department of Internal Medicine, Frankfurt (Oder) General Hospital, Frankfurt/Oder, Germany; 6 Department of Hematology, Oncology and Palliative Care, Vivantes Clinic Neukoelln, Berlin, Germany; 7 Department of Bone Marrow Transplantation, West German Cancer Center, University Hospital Essen, University of Duisburg-Essen, Essen, Germany; 8 Department of Hematology and Oncology, Campus Fulda, Philipps-University Marburg, Fulda, Germany; 9 Department of Hematology and Oncology, University Hospital Oldenburg, Oldenburg, Germany; 10 Department of Hematology, Oncology, and Tumor Immunology, Charité - Universitätsmedizin Berlin, Berlin, Germany; 11 Department of Hematology and Oncology, Helios Klinikum Aue, Aue, Germany; 12 Department of Internal Medicine, Infectious Diseases, University Hospital Frankfurt, Goethe University Frankfurt, Frankfurt am Main, Germany; 13 Department of Hematology, Oncology and Palliative Care, Department of Internal Medicine, Evangelisches Klinikum Bethel, Bielefeld, Germany; 14 Department III of Internal Medicine, Hematology, Oncology and Hemostaseology, Südharzklinikum, Nordhausen, Germany; 15 Department of Hematology, Oncology & Palliative Care, Klinikum Neuperlach, Munich, Germany; 16 Department of Hematology and Oncology, Red Cross Hospital Munich, Munich, Germany

**Keywords:** Central venous catheter, Catheter infection, CRBSI, CLABSI, Neutropenia, Cancer

## Abstract

Cancer patients frequently require central venous catheters for therapy and parenteral nutrition and are at high risk of central venous catheter–related infections (CRIs). Moreover, CRIs prolong hospitalization, cause an excess in resource utilization and treatment cost, often delay anti-cancer treatment, and are associated with a significant increase in mortality in cancer patients. We therefore summoned a panel of experts by the Infectious Diseases Working Party (AGIHO) of the German Society of Hematology and Medical Oncology (DGHO) and updated our previous guideline on CRIs in cancer patients. After conducting systematic literature searches on PubMed, Medline, and Cochrane databases, video- and meeting-based consensus discussions were held. In the presented guideline, we summarize recommendations on definition, diagnosis, management, and prevention of CRIs in cancer patients including the grading of strength of recommendations and the respective levels of evidence. This guideline supports clinicians and researchers alike in the evidence-based decision-making in the management of CRIs in cancer patients.

## Introduction

Cancer patients frequently require central venous catheters (CVCs) for cancer treatment, blood transfusion, and parenteral nutrition. However, cancer patients are at particular risk of infections including CVC-related infections (CRIs) due to disease- and treatment-related immunosuppression. According to current estimates, more than 5 million CVCs are inserted in the USA annually and similar rates have been reported for European countries [1–4]. The frequency of resulting central line–associated bloodstream infections (CLABSIs) in cancer patients is estimated at 0.5–10 per 1000 CVC-days. The associated mortality ranges from 12 to 40% depending on several factors, including patient comorbidities, CVC type, and microorganism causing the infection [2, 5–8]. Importantly, up to 70% of all CRIs may be preventable with current evidence-based strategies [9]. Several institutions and public authorities have issued comprehensive guidelines on CRIs such as the German Commission for Control and Prevention of Infections (KRINKO). These recommendations and guidelines may include obligatory measures and have high normative value but are not specifically targeted at cancer patients. The guideline presented here is based on our previous guideline [10] that summarizes current data on epidemiology, diagnosis, treatment, and prevention of CRIs in cancer patients to guide clinicians and identify areas of uncertainty.

## Methods

We assigned subtopics of this guideline to a panel of 20 experts in the field of internal medicine, hematology and oncology, infectious diseases, infection control and hospital epidemiology, and critical care medicine. We then conducted independent literature searches of the PubMed, Medline, and Cochrane databases using combinations of the following search terms: central venous catheter infection, central venous catheter-related bloodstream infection, central venous catheter-associated bloodstream infection, cancer, neutropenia, definition, pathogenesis, pathogens, epidemiology, incidence, risk factors, diagnosis, treatment, management, surveillance, education, and prevention. The consensus process was carried out in e-mail-, telephone-, video-, and meeting-based discussion groups. The strength of each recommendation and the grade of evidence were adapted to the criteria of the European Society for Clinical Microbiology and Infectious Diseases (ESCMID; Table 1) [11]. The presented guideline replaces our previous guideline [10] and was approved by the assembly of the members of the Infectious Diseases Working Party (AGIHO) of the German Society of Hematology and Medical Oncology (DGHO) on March 23, 2018, and again after updating the recommendations and references on May 7, 2020, as a video conference. All authors approved the final version of the manuscript and the recommendations before submission.

**Table 1:** Categories of evidence levels used in this guideline

Category, grade	Definition
Strength of recommendation	
A	Strongly supports a recommendation for use
B	Moderately supports a recommendation for use
C	Marginally supports a recommendation for use
D	Supports a recommendation against use
Quality of evidence	
I	Evidence from at least one properly designed randomized, controlled trial
II*	Evidence from at least one well-designed clinical trial, without randomization; from cohort or case-controlled analytic studies (preferably from > 1 center); from multiple time series; or from dramatic results of uncontrolled experiments
III	Evidence from opinions of respected authorities, based on clinical experience, descriptive case studies, or reports of expert committees

*Added index: r: Meta-analysis or systematic review of randomized controlled trials. t: Transferred evidence, that is, results from different patients’ cohorts, or similar immune status situation. h: Comparator group is a historical control. u: Uncontrolled trial. a: Published abstract (presented at an international symposium or meeting)

## Results

### Definitions

Based on clinical symptoms and laboratory findings, localized infections of CVCs such as exit-site infections, tunnel infections, and port–pocket infections are distinguished from CLABSIs or catheter-related blood stream infections (CRBSI). However, the definitions of CLABSI or CRBSI are not interchangeable, as criteria vary substantially between the two definitions [1, 20]. Importantly, the Infectious Diseases Society of America (IDSA) definition of CRBSI and the US Centers for Disease Control and Prevention (CDC) definition of CLABSI do not specifically target cancer patients and lack specificity in this particular patient population [1, 20–27]. As the CDC definition of CLABSI was shown to overestimate the rate of CVC-derived bacteremia in cancer patients, the concept of mucosal barrier injury (MBI) laboratory-confirmed bloodstream infection (LCBI) was proposed [20]. This surveillance definition intends to identify a subset of bacteremia in cancer patients, which is likely to be related to mucosal barrier injury with bacterial translocation from the gastrointestinal tract and not related to infection of a CVC. However, the criteria for MBI-LCBI are restricted to specific subsets of cancer patients and specific microorganisms and might have limited applicability in clinical practice [28, 29]. In this regard, a recent retrospective review of 250 patients in a Japanese academic hospital identified 44 patients during a 47-month period with CLABSI, of which about half (45.5%) met the definition of MBI-LCBI and 24 (54.5%) were classified as non-MBI-LCBI [30]. Similarly, Chaftari and colleagues reviewed 149 cases of CLABSI at their institution, of which 70 (47%) had definite CRBSI. Even though CRBSI was more common in patients with non-MBI-LCBI, about one in five patients with MBI-LCBI (18%) had definitive CRBSI [22]. Thus, the use of MBI-LCBI criteria in cancer patients might be useful for surveillance purposes, but might have limited applicability to everyday practice. We thus recommend against the use of CLABSI for the definition of CRIs in cancer patients (DII). To account for the specific characteristics of cancer patients, we recommend the distinction between “definite,” “probable,” and “possible” CRBSIs as proposed in 2012 and outlined in Table 2 [10, 31–34] (AII).

**Table 2:** Diagnostic criteria for CVC-related bloodstream infections (CRBSI)

Diagnosis	Criteria (I)	Criteria (II)
Definite CRBSI	Growth of same pathogen from blood culture of peripheral vein and from culture of CVC tip	± in vitro susceptibility testing results in the same resistance pattern (AI) [12]
	Growth of same pathogen from blood culture of CVC and from blood culture of peripheral vein	And DTTP ≥ 2 h (AIIt) or, for quantitative blood cultures, a ≥ 3-fold greater colony count of pathogens grown from blood culture of CVC than the colony count from a peripheral vein (AIIt) [1, 12, 13]
		DTTP >2 h is inaccurate to rule out CRBSI in patients with detection of *S. aureus* [14, 15] or *Candida* spp. [16–19] (DIIt)
Probable CRBSI	Growth of the same pathogen from blood culture of CVC and from blood culture of peripheral vein	And no criteria for definitive CRBSI
		And detection of coagulase-negative *Staphylococcus* spp., *S. aureus*, or *Candida* spp.
		And exclusion of other infection sites (BIII)
Exit-site infection	Clinical signs of infection ≤ 2 cm from the CVC exit	And BSI without criteria for definitive CRBSI (BIII)
Tunnel infection (Hickman and Broviac catheter)	Clinical signs of infection > 2 cm from CVC exit site along the subcutaneous part of CVC	And BSI without criteria for definitive CRBSI (BIII)
Pocket infection (implanted port system)	Clinical signs of infection of subcutaneous pocket	And BSI without criteria for definitive CRBSI (BIII)
Possible CRBSI		
CVC colonization	Growth of pathogen from CVC tip (> 15 CFU in semiquantitative/> 100 CFU in quantitative culture)	And clinical or laboratory signs of infection (e.g., leukocytosis or elevated C-reactive protein)
		And no BSI (BIII)
	Pathogen detected in blood culture that is typically causing CRI (*S. epidermidis*, *S. aureus*, *Candida* spp.)	And no other focus identified (BIII)
	Remission of fever in < 48 h after CVC removal	And no other focus identified (BIII)

*CRBSI*, catheter-related bloodstream infection; *BSI*, bloodstream infection; *CFU*, colony forming unit; *CVC*, central venous catheter; *DTTP*, differential time to positivity of CVC blood culture and peripheral blood culture

### Pathogenesis and risk factors

In short-dwelling catheters (< 14 days), colonization of CVCs via migration of skin microorganisms resulting in extraluminal spread of bacteria along the outer surface of the catheter predominates as pathomechanism of infection [35]. Within 24 h of CVC placement, the interior surface of the catheter may be covered with a biofilm embedding bacteria and fungi. Consequently, colonization and infection via catheter hubs and, less frequently, via infusion solutions resulting in intraluminal spread of microorganisms are more common in longer-dwelling CVCs (≥ 14 days) [1, 35, 36]. Risk factors for catheter-related infections include a high level of skin colonization at the insertion site and the catheter hub as well as the administration of blood products and total parenteral nutrition [7, 21, 37, 38]. Unsurprisingly, patients requiring more than one CVC are at higher risk of CRI [39]. Among cancer patients, patients with hematological malignancies are at higher risk for CRIs compared with patients with solid tumors and the risk of infection is higher in patients with aggressive hematological malignancy such as leukemia and high-grade lymphoma, compared with patients with less aggressive malignancies [5, 40–42]. Neutropenia is a major independent risk factor for CRIs, and neutropenic patients with bloodstream infections are at higher risk of mortality compared with non-neutropenic patients [43–46]. Interestingly, neutropenia at the time of CVC insertion had no association with rates of definitive or probable CRBSI comparing matched neutropenic with non-neutropenic patients in a recent analysis of 806 patients [47]. Although patients undergoing allogeneic or autologous hematopoietic stem cell transplantation (HSCT) are commonly neutropenic, HSCT might further increase the risk of CLABSI and CRBSI independent of the impact of neutropenia. In a recent retrospective study by McDonald and colleagues on 352 patients undergoing allogeneic HSCT, the use of a matched unrelated donor (MUD) and/or haploidentical donor and the use of an ablative conditioning regimen were independently associated with development of CLABSI on multivariate analysis [48]. Thrombosis, even when detectable only by ultrasound screening, was shown to be a risk factor for CRIs, and infection of the catheter in turn promotes thrombosis [49–51]. Other risk factors include male gender, disease stage, age, and reduced performance status [52, 53]. Of note, rates of CRI also depend on the devices used, and the risk of CRI differs between cuffed tunneled CVCs, subcutaneous implanted ports, peripherally inserted CVCs (PICCs), and percutaneous non-cuffed or tunneled CVCs [21]. Reported rates of CRIs in implantable and tunneled catheters are lower compared with rates in non-tunneled catheters [21, 54, 55]. PICCs have become more common for patients requiring long-term venous access, and reported rates of CRIs in several studies and one recent meta-analysis suggest a lower or comparable risk of CRI compared with CVCs [56–63]. In addition, timing and procedure of CVC placement also might influence CRI rates, as catheter placement by interventional radiologists was reported to be associated with less complications including CRIs, than surgically placed catheters [64]).

### Epidemiology

The incidence of CRI complications including CRBSI in cancer patients is highly dependent on the studied patient population, the setting, and the definitions used. As a consequence of the high variability of the studies, the rate of CRIs in cancer patients reported in the literature varies between 9 and 80% [65]. A recent large retrospective study analyzed registry data (SEER-Medicare) of more than 35,000 adult cancer patients above the age of 65 years with long-term catheters (mainly port catheters) [53]. The authors reported an overall incidence of CRIs of 16–31% in patients with long-term CVCs and a two- to five-fold risk of CRIs compared with matched controls without CVC, suggesting an important impact of catheterization on the incidence of CRIs [53].

Surveillance and cohort studies in cancer patients report CRBSI/CLABSI rates of 1.05–14.4 per 1000 CVC-days [6, 41, 66–68]. A recent pooled analysis of 1194 cancer patients derived from the German SECRECY registry and a prospective randomized trial testing an antimicrobial dressing in neutropenic cancer patients (COAT-trial) used the definitions of definite CRBSI and definite plus probable CRBSI, reporting an incidence of 2.7 and 6.7 for definite CRBSI and definite plus probable CRBSI per 1000 catheter-days, respectively [32]. Using the less stringent CDC definition, a CLABSI rate of 2.9–6.3 per 1000 CVC-days was reported in a recent randomized controlled trial in adult cancer patients testing different skin disinfectant solutions (alcohol-based solutions with or without octenidine) [37, 69]. Higher rates were reported in studies focusing on neutropenic patients and patients receiving autologous or allogeneic hematopoietic stem cell transplantation, with CRBSI/CLABSI rates up to 24.3 per 1000 neutropenic days in one study [48, 70–72]. In a retrospective study on patients in an outpatient transplant unit at an academic tertiary center, the cumulative incidence of CLABSIs within 100 days after allogeneic stem cell transplantation (SCT) was 9%, with the majority (67%) occurring within the first 30 days after transplant [48]. The German ONKO-KISS surveillance registry reported CLABSI incidence of 4.6 and 3.4 per 1000 CVC-days in autologous and allogeneic HSCT recipients, respectively, in 2019. During neutropenia, these rates increased to 10.6 and 5.9 per 1000 CVC-days in patients after autologous and allogeneic HSCT, respectively [73].

### Pathogens

The distribution of pathogens causing CRIs in cancer patients depends on the population studied and the definition of CRIs applied. Overall, coagulase-negative staphylococci (CoNS) are the most commonly detected bacteria in cancer patients with CLABSI, followed by other Gram-positive bacteria such as *Staphylococcus aureus*, Enterococci, and Streptococci [44, 74–77]. Accordingly, Schalk and colleagues recently reported a large multicenter cohort of 3000 cases of definitive and probable CRBSI in cancer patients from registry and trial data and found a similar distribution of pathogens as in smaller single-center studies with CoNS as the most common causative pathogens for CRBSIs [32]. The frequency of Gram-negative bacteria such as *Escherichia coli*, *Pseudomonas aeruginosa*, and *Klebsiella* spp. varies between studies within the range of 20–27% [28, 44, 74, 75]. Recent longitudinal studies suggest a shift from the predominance of Gram-positive to Gram-negative bacteria causing CRBSI in cancer patients in more recent periods [77, 78]. A recent retrospective study compared two cohorts of cancer patients from 1999 to 2000 and 2013 to 2014, and found Gram-negative organisms as predominant etiologic bacteria in the latter period contributing to 41% of the CRBSI [79]. In addition, Gram-negative bacteria are more commonly found as microorganisms causing blood stream infections (BSI) in neutropenic patients, compared with non-neutropenic patients [28, 80]. This is likely due to bacterial translocation of gut organisms frequently causing BSI rather than CRI in neutropenic patients [81]. Increasing rates of antibiotic resistance including multi-drug resistance (MDR) have been reported worldwide in the last decade also in studies focusing on cancer patients [82–84]. Therefore, local epidemiology and resistance patterns as well as known individual colonization with resistant pathogens should be considered factors for the choice of empiric antibiotic therapy in patients with CRBSI [45, 46].

*Candida* spp. have been reported in 2–13% of patients with CRBSI [31, 44, 74], and polymicrobial cultures were reported in 11–30%, with the incidence again depending on the definition of CRBSI used [31, 74, 85, 86]. Using the definition of definite CRBSI and probable CRBSI, CoNS, Gram-negative bacteria, and *Candida* spp. were reported in 73%, 15.5%, and 1%, respectively, in a recent multicenter study for both definite CRBSI and probable CRBSI combined in cancer patients [31]. Of note, the predominance of Gram-negative bacteria that had been described in other recent cohort studies using less cancer-specific definitions was not observed with the use of the definition of definite plus probable CRBSI [31, 32].

### Diagnosis

Diagnostic procedures for the detection of CRIs should be initiated in patients with any type of CVC (conventional CVCs, PICCs, implantable CVCs, etc.) upon clinical signs and symptoms of infection and without any other apparent source of infection. Symptoms may include local signs such as erythema, swelling and pain or more frequently systemic signs such as fever and hypotension, or a combination of both. In all patients, a thorough physical examination should be performed, complemented by microbiological testing (blood cultures) and imaging according to current guidelines [45, 87].

#### Diagnostic procedures for suspected CRBSI

In patients with suspected CRBSI, at least two pairs of blood cultures with adequate quantity of blood (≥ 10 ml depending on the culture flask used) should be taken simultaneously, one pair from a peripheral vein and one pair from the CVC [1, 88–90]. Samples should be drawn before the administration of antibiotics and under sterile precautions to avoid contamination. Studies sampling all lumens in multi-lumen CVCs indicate that colonization might be detectable only in one of several lumens, and therefore, blood cultures from all lumens should be sampled [91–94]. However, the higher detection rate needs to be carefully balanced against the potential harm caused by the withdrawal of large blood volumes.

Quantitative or semiquantitative blood cultures taken simultaneously from the CVC and a peripheral vein with a colony count ratio of 3:1 to 10:1 of the same microorganism species are considered indicative of CRBSI [12, 36, 65]. However, the method of testing for quantitative blood cultures is time consuming, elaborate, and expensive, and the availability is therefore limited and not a clinical routine in most microbiological laboratories [65].

The differential time to positivity (DTTP), defined as > 2 h earlier positivity of CVC-drawn versus peripheral blood cultures detecting the same pathogen during automated incubation, has been reported as a sensitive and specific diagnostic marker for CRBSI in patients with short- and long-term CVCs [95, 96]. The method was studied in intensive care unit (ICU) patients and cancer patients including neutropenic patients and recipients of allogeneic HSCT [65, 74, 95–97]. A DTTP > 2 h has been shown to be predictive for CRBSI with reported sensitivity and specificity ranging from 72 to 100% and a negative predictive value of 91–92% [74, 92, 95, 96]. DTTP was therefore proposed as a useful diagnostic tool particularly to prevent unnecessary CVC removal in patients with limited intravenous access options [65, 74]. Recent studies evaluating the 2-h cutoff DTTP for patients with candidemia [16–19] and *S. aureus* bacteremia [14, 15] showed insufficient test performance to diagnose or exclude CRBSI in patients with detection of these pathogens. Therefore, the use of DTTP in patients with candidemia and *S. aureus* bacteremia is not recommended for diagnosis or exclusion of CRBSI and as a decision tool for CVC preservation.

In patients without possibility of blood culturing because no blood can be aspirated via the catheter, sampling the internal catheter surface in situ by endoluminal brushing may be useful [91, 92]. However, the technique is not widely available and might underestimate CRI in short-dwelling CVCs where external surface colonization plays an important role. Moreover, endoluminal brushing might carry the risk of inducing bacteremia in patients with colonized catheter and is not recommended for routine diagnostics.

If the catheter is removed in case of suspected CRI, the catheter tip should be cut to a length of ∼ 5 cm and placed in a sterile dry container for transport. Standard methods for microbiological diagnosis of CRI after CVC removal have previously been reviewed [13, 98].

#### Diagnostic procedures for suspected localized CRI

**Fig. 1:**
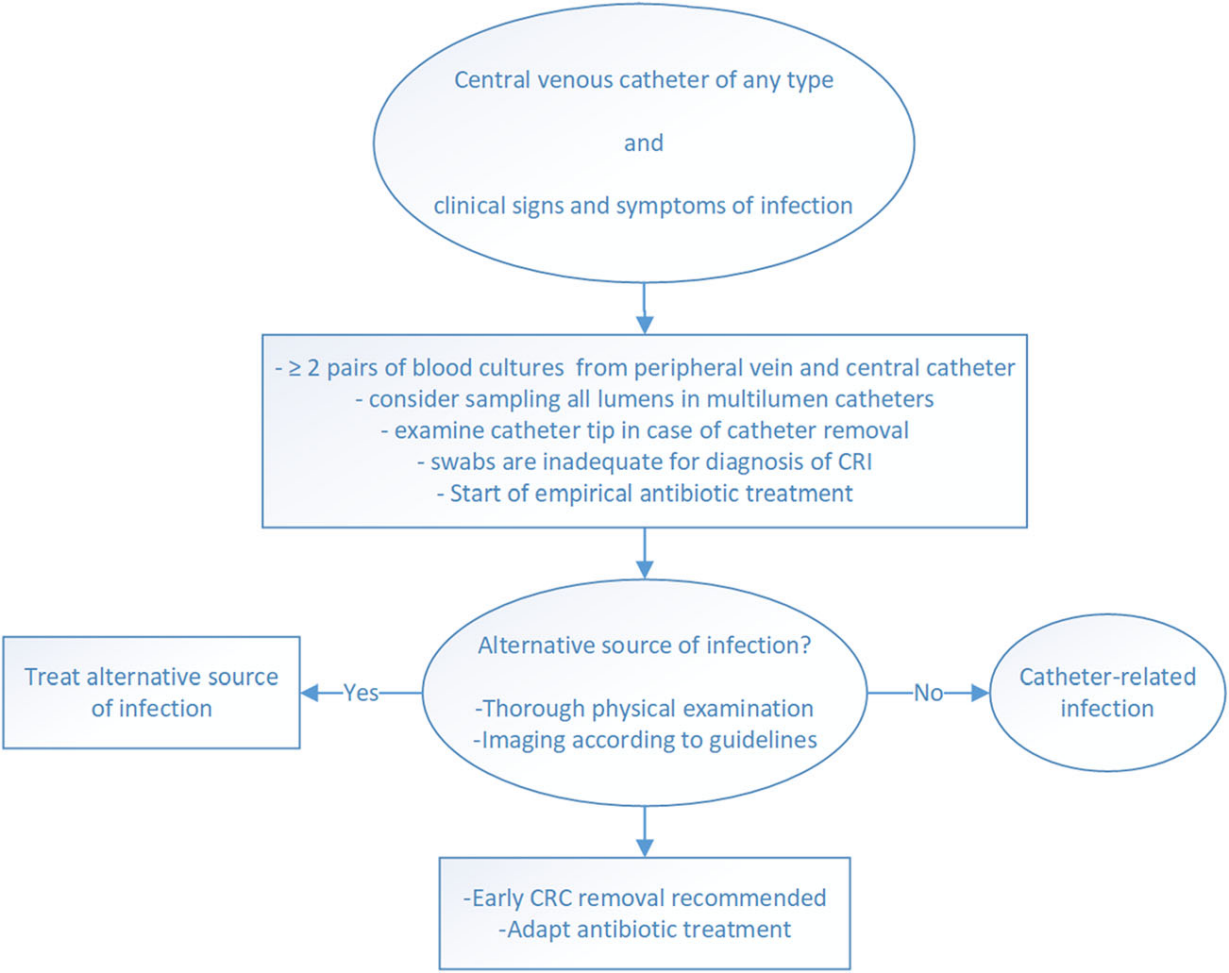
Diagnostic procedures in case of suspected catheter-related infection (CRI)

Local CRI as exit-site infections and tunnel infections should be suspected based on clinical signs and symptoms. Some authors recommend taking a swab for culture and staining in case of secretion at the exit site of the CVC [1, 88]. Nonetheless, skin swabs do not allow for a reliable differentiation between colonizing and pathogenic organisms, even in case of purulent secretion at the exit site, and have limited validity in patients with suspected CRI [35, 88, 99]. In a recent study, cultures of skin swabs from the skin overlying reservoir ports and from the insertion site and hubs of tunneled catheters had low sensitivity and specificity (23–45% and 60–63%, respectively) for the prediction of CRBSI, defined as isolation of the same microorganism in both the colonized CVC and at least one peripheral blood culture obtained 1 week before or after catheter withdrawal [100]. Therefore, skin swabs have limited validity to confirm or rule out local CRI and are not recommended in clinical routine. Diagnostic procedures in case of suspected CRI are summarized in Fig. 1, and recommendations are summarized in Table 3.

**Table 3:** Standard procedures in the diagnosis of CVC-related infections (CRI)

Before CVC removal	
• Rule out other possible sources of infection by clinical examination and imaging procedures, if necessary.	
• Inspect the CVC insertion site or pocket or tunnel for signs of local infection. Palpate the pocket or tunnel.	
• Do not take skin swabs as they have limited validity to confirm or rule out local CRI in clinical routine (DIII).	
• Take one pair of blood cultures (aerobic and anaerobic) from a peripheral vein and one from the CVC for microbiological evaluation (AIIt).	
• In case of multi-lumen CVC, draw separate blood cultures from each lumen (BII).	
• Determine the DTTP between the CVC and peripheral blood culture sample (for pathogens other than *S. aureus* or *Candida* spp.) (AIIt).	
• Do not use DTTP in patients with candidemia and *S. aureus* bacteremia for diagnosis or exclusion of CRBSI and as a decision tool for CVC preservation (DIIt).	
• Do not use endoluminal brushing for routine diagnostics (DIII).	
After CVC removal	
• Perform a microbiological examination of the CVC tip (AIIt).	

*DTTP*, differential time to positivity; *CVC*, central venous catheter

### Prevention

Recommendations for the prevention of CRI are summarized in Table 4.

**Table 4:** Prevention of CVC-related infections (CRI)

• Avoid unnecessary catheterization and remove CVCs no longer required (AIIt).	
• Implement education programs and bundles for nurses and physicians including continuous surveillance and feedback to reduce the incidence of CRIs (AIIt).	
• Compliance with hygiene principles during insertion and standardized aseptic placement and handling of the catheter help to avoid CRIs (AIIt).	
• Alcoholic chlorhexidine solution with polyvidone-iodine solutions or octenidine/propranolol solutions should be used for disinfection of the catheter insertion site (AI).	
• Ultrasound-guided placement ultrasound may reduce the rate of mechanical complications (AIIt) and the number of cannulation attempts with a possible impact on the incidence of CRI (CIIt).	
• Avoid femoral catheterization (DIIt).	
• Catheter fixation using sutureless devices might reduce the risk of CRI (BI).	
• Cover insertion site using sterile gauze or transparent film (AI).	
• Replace CVC gauze dressings every 2 days and transparent dressings once weekly, unless there are signs of local contamination, inflammation, or detachment (BI).	
• Chlorhexidine-containing dressings, preferably transparent chlorhexidine-impregnated gel dressings, may be used alternatively as they might reduce the risk of CRIs (BI).	
• Antimicrobial-impregnated CVCs may be useful in patients with long-term CVCs in case of persisting high rate of CRI despite implementation of educational programs and appropriate CVC bundles (CIIt).	
• Antibiotic lock solutions should be limited to persisting high baseline rates of CRI in high-risk patients with long-term catheters (BI).	
• Do not screen for CRIs in asymptomatic patients by routine withdrawal of blood cultures (DII).	
• Do not apply systemic prophylactic antibiotic treatment prior to catheter insertion (DI).	
• Do not apply topical antibiotic ointments for reducing staphylococcal colonization at the CVC insertion site (DII).	
• Routine replacement does not reduce the incidence of CRI (DI).	

*CRI*, catheter-related infection; *CRBSI*, catheter-related bloodstream infection; *CVC*, central venous catheter

#### Education, bundles, and surveillance

Avoiding unnecessary catheterization and prompt removal of no longer required CVCs are effective measures to reduce CRBSI, in particular in longer-dwelling catheters. Thus, institution-wide standards such as daily audits to assess whether each CVC is still needed are strongly encouraged. The use of bundles for the prevention of CRIs, including prospective consistent surveillance of CRI rates; education, instructions, and surveillance of hand hygiene; aseptic catheterization; and handling of CVCs, effectively reduces CRI rates and is recommended [101–107]. These bundles may also include recommendations on appropriate nursing staff levels and the designation of designated trained personnel for placement and handling of CVCs, as studies indicate an impact of both factors in lowering CRI rates [108, 109]. These bundles particularly stress the need for hand hygiene and disinfection of catheter access sites prior to manipulation. In addition to these measures, close collaboration between the primary caring oncologist and infection control/microbiology and ICU physicians is essential in the prevention of CRIs.

Several institutions have issued recommendations on prevention of CRI that are largely based on studies not exclusively focusing on cancer patients. Nonetheless, recent smaller studies in cancer patients show similar results in terms of the effectiveness of education and surveillance methods to prevent CRIs in cancer patients [71, 110–113]. In a recent study, Chaftari and colleagues conducted a quality improvement project focusing on simultaneous peripheral and central blood culture drawing with accurate source labeling in cancer patients. After staff education and monitoring, the average blood culture source labeling improved from a baseline of 48 to 70% and identification of the CVC as source of bacteremia was successful in 88% of cases compared with 36% at baseline (*P* = 0.0003), suggesting that education measures are equally effective in cancer patients for the prevention of CRIs [110].

Screening for CRIs in asymptomatic patients by routine withdrawal of blood cultures is not recommended, as weekly or even daily blood culturing is not effective in the earlier detection of CRI in cancer patients including HSCT recipients [114–117].

#### Sterile precautions, skin antisepsis, and CVC replacement

CVC insertion should be attempted under maximal sterile barrier precautions including sterile gown, gloves, and cap and using a large sterile drape and implementing aseptic bundles as they decrease the risk of CRI [102, 118].

Ultrasound (US)-guided central venous catheterization is associated with lower rates of pneumothorax and other mechanical complications and may reduce the number of cannulation attempts [119–121]. Although the impact on the incidence of CRI is less clear, central venous cannulation using US is recommended for the avoidance of mechanical complications and the reduction of cannulation attempts with a possible positive impact on the incidence of CRI [119, 122].

Cutaneous antisepsis using > 0.5% chlorhexidine alcohol–based solution (CBA) results in lower rates of CRBSI compared with 10% polyvidone-iodine or 70% alcohol-only solutions as shown in studies and meta-analyses, although alcoholic polyvidone-iodine solutions (A-PVP) or 70% propranolol may be safe alternatives in case of allergy or intolerance to chlorhexidine [123–131]. Accordingly, the use of antiseptic agents containing only alcohol is not recommended for insertion of CVCs by institutional guidelines such as the KRINKO [27]. Several studies have analyzed and tested the sequential or parallel application of disinfectants such as CBA and A-PVP [69, 132–134]. For instance, sequential CBA with A-PVP was superior to either of the regimens alone in terms of a lower rate of CVC tip colonization in 119 patients on normal wards and ICUs [134]. A recent meta-analysis analyzing several studies concluded that sequential antiseptic use reduces the likelihood of CVC colonization compared with the use of either agent alone, although the impact on the risk of CRBSI risk is less clear [132]. Therefore, combinations of CBA with A-PVA or octenidine/propranolol solutions are alternatives for cutaneous antisepsis. If necessary, the insertion site should be cleaned prior to disinfection.

Daily chlorhexidine (CHX) bathing reduces the incidence of CRBSI in patients in ICUs. However, the impact of CHX bathing on CRBSI in cancer patients has not been well studied and is therefore not recommended in clinical routine [135, 136].

Although longer dwelling times of CVCs increase the risk of CRBSI, routine replacement did not reduce the incidence of CRBSI in adult ICU patients [137, 138]. A large cohort analysis examined CVC duration to predict CRBSI in 1194 cancer patients and failed to determine an optimal cutoff time point at which a prophylactic CVC exchange would prevent CRBSI [33]. Thus, routine replacement of CVC is not recommended [27]. Infusion and tubing systems should be replaced as previously recommended [10, 139].

#### CVC site dressing and anti-infective caps

Sterile gauze or transparent film should be used as dressing to cover the CVC insertion site [25]. CVC gauze dressings should be replaced every 2 days, transparent dressings once weekly, unless there are signs of local contamination, inflammation, or detachment [1, 25, 27, 140]. Whether the use of gauze or transparent dressings is preferable in terms of lower CRI rates was addressed in two recent meta-analyses [141, 142]. Dang and colleagues reported that transparent dressings were associated with a lower risk of CRBSI [142]. In contrast, an analysis of 22 studies did not find sufficient evidence for a difference in the rate of CRBSI between different non-impregnated dressings [141]. Similarly, a recent systematic review in HSCT recipients found no difference between the type of dressing and the incidence of CRBSI [143]. Therefore, gauze, tape, or transparent polyurethane dressings can be recommended for CVC site insertion dressing without clear preference of one over the other.

CHX-impregnated dressings were tested in cancer and ICU patients in randomized trials showing a reduction in CRI rates compared with standard dressings, and this finding could be confirmed in a recent systematic review and meta-analysis [31, 144–148]. A recent multicenter randomized trial studied the use of dressings containing CHX-containing gel pads compared with standard non-impregnated dressings in 613 neutropenic cancer patients using the stringent definition of definite CRBSI and probable CRBSI [10, 31]. Although difference in the primary end point of definite CRBSI after 14 days did not meet statistical significance, both definite plus probable CRBSI (dpCRBSI14) after 14 days and overall definite plus probable CRBSI (dpCRBSI) were significantly less frequent in the CHX group compared with control. Rates of dpCRBSI14 were 6.5% (20/307) in the CHX group compared with 11% (34/306) in the control group (*P* = 0.047), and dpCRBSI occurred in 10.4% (32/307) and 17% (52/306) in the CHX and the control groups, respectively (*P* = 0.019). Moreover, CHX dressings were well tolerated as the frequency of dressing intolerance with cutaneous and soft tissue abnormalities at the contact area was similar in both groups (12.4% and 11.8%; *P* = 0.901) [31]. Therefore, the use of CHX-containing dressings might be helpful for the prevention of CRIs in cancer patients, preferably transparent CHX-impregnated gel dressings, as CHX sponges might conceal the insertion site and increase the risk of dressing detachment [146, 147]. As CRIs are often preceded by hub colonization, disinfectant caps have been tested in smaller observational trials and might be a promising approach to reduce the incidence of CRIs in cancer patients [149, 150].

#### Choice of CVC, sutureless devices, and impact of catheterization site

Randomized trials and meta-analyses have shown no difference in risk of CRIs between single- or multiple-lumen CVC, and therefore, a preferred use of single-lumen catheters is not supported [151–153]. The use of sutureless devices was found to reduce the risk of CRI in a randomized study and in two meta-analyses accounting for multiple treatments [141, 142, 154].

The association of catheterization site and CRI has been studied in several earlier studies and meta-analyses [155–158]. Overall, the insertion in femoral sites has been associated with a higher risk of infections and thrombotic complications compared with subclavian and internal jugular CVCs. In a recent large randomized study comparing different insertion sites in more than 3000 adult patients on ICUs, the risk of an event in composite outcome of CRBSI and symptomatic deep-vein thrombosis was significantly higher in the femoral group compared with a subclavian approach [159]. In accordance, two meta-analyses concluded that the use of femoral catheters increases the risk of CRBSI compared with internal jugular and subclavian catheters [160, 161]. Therefore, femoral catheterization should be avoided. Subclavian insertion might be preferable over internal jugular, as colonization risk and risk of CRBSI might be slightly lower at subclavian sites [159–161]. However, in a recent retrospective single-center analysis on 56 patients undergoing allogeneic HSCT, there were no differences in the frequency of CLABSI, deep-vein thrombosis, pneumothorax, and catheter lumen obstruction between catheters inserted into either internal jugular or subclavian vein [162]. Of note, in larger studies, insertion at the subclavian site was associated with higher risk of mechanical complications, in particular pneumothorax and hemorrhage [159, 163, 164].

#### Antimicrobially impregnated CVCs

Multiple studies and meta-analyses showed a reduction in catheter colonization by use of antiseptic-coated CVCs, usually using CVCs coated with CHX, silver sulfadiazine, or both [165–169]. However, a reduction in CRBSI rates by the use of antimicrobial CVCs was not consistently found. The use of minocycline/rifampicin or miconazole/rifampicin-coated catheters resulted in a reduced incidence of CRI in the majority of trials performed, and despite initial concerns, no higher incidence of antibiotic resistance was observed with the use of antibiotic CVCs [170–177]. Notably, in the largest trial exclusively including cancer patients, the intervention was tested in long-term catheters with catheters used for more than 2 months [173]. In conclusion, the use of antimicrobial-impregnated CVCs may be useful in patients with long-term CVC in case of persisting high rate of CRI despite implementation of educational programs and appropriate CVC bundles.

#### Systemic and topical antibiotic prophylaxis

Systemic antibiotic prophylaxis before CVC insertion does not reduce CRIs in cancer patients [178]. A recent single-center study on fluoroquinolone prophylaxis in recipients of autologous HSCT during neutropenia suggested a benefit in reduction of CRIs. However, this was a retrospective study that used the incidence of CLABSI rather than CRBSI as primary endpoint [179]. Therefore, systemic antimicrobial prophylaxis before CVC insertion is not recommended for the prevention of CRIs. Accordingly, as topical antibiotics have not been shown to reduce risk of CRIs and might increase the risk of antibiotic resistance, the use of topical antibiotics is not recommended for the prevention of CRI [180].

#### Antimicrobial lock solutions for prevention of CRIs

Heparin lock solutions are commonly used, although saline solution might be a safe alternative as it proved non-inferior in terms of functional problems and CVC-related bacteremia in a randomized trial including 802 cancer patients with totally implantable venous access devices [181]. Taurolidine-citrate-heparin did not result in significantly less CVC hub colonization and CRBSI than placebo in neutropenic hematologic patients in a prospective multicenter trial involving 150 patients with non-tunneled CVCs [182]. Ethanol 70% lock solution has been studied in several randomized trials including heterogenous study populations with conflicting results. In a placebo-controlled randomized trial including 64 hematologic patients with cuffed subclavian Hickman catheters, daily administrations of ethanol locks effectively reduced the incidence of CABSI from 0.60/100 catheter-days in the ethanol group to 3.11/100 catheter-days in the control group [183]. Similarly, 2-hour ethanol locks once weekly resulted in a reduced CABSI incidence in a randomized trial in 307 pediatric oncology patients, predominantly less Gram-positive CLABSIs [184]. In contrast, two recent randomized trials, one of those using the more stringent CRBSI definition, failed to show a significant reduction of CRIs in cancer patients by use of ethanol locks [185, 186].

As CRIs are in most cases preceded by CVC colonization, antibiotic lock solutions were tested as means of preventing bacterial colonization and subsequent CRI. Instillation of vancomycin resulted in lower rates of CVC hub colonization with Gram-positive bacteria and subsequent bacteremia during neutropenia in a randomized single-center study including 120 cancer patients [187]. Three meta-analyses suggested a reduction of CRI by antibiotic locks (ALT) [178, 188, 189]. However, the studies included were heterogenous as the populations studied were in part pediatric cancer patients or hemodialysis patients and the treatment protocols varied substantially. Therefore, ALT should be limited to persisting high baseline rates of CRI in high-risk patients with long-term catheters, and the potential beneficial effects of ALT must be balanced against the potential for allergic reactions, toxicity, and emergence of antimicrobial resistance.

### Management

Recommendations for the management of CRI are summarized in Table 5 and in Table 6.

**Table 5:** Management of CVC-related infections (CRI)

• Remove the CVC in patients with CRI whenever possible (AIIt).	
• CVC removal is necessary in patients with tunnel and pocket infections (BIII).	
• In severely thrombocytopenic patients with limited venous access, the risk of catheter reinsertion should be carefully weighed against the risk of patient deterioration and prolongation of the infection (BIII).	
• CVC exchange over a guidewire is not recommended as an alternative approach to removal (DIII).	
• Early CVC removal is particularly encouraged in patients with deteriorating clinical state, sepsis, or septic shock and in case of severe complications such as endocarditis, septic thrombosis, abscess formations, or osteomyelitis (BIII). In case of preserved catheter, prompt removal is warranted in any case of clinical deterioration or continued positive blood cultures 72 h after initiation of therapy in spite of appropriate antimicrobial therapy.	
• Early CVC removal is always recommended in patients with CRBSI due to *S. aureus* (AIIt).	
• Early catheter removal is always recommended in patients with CRBSI due to *Candida* spp. (AIIt).	
• Catheter removal within 48–72 h is recommended in case of CRBSI caused by Gram-negative bacteria (BIIt).	
• Preservation of CVC may be initially attempted in clinically stable patients in the presence of coagulase-negative staphylococci or *Corynebacterium jeikeium* (BIIt).	
• An antimicrobial lock technique may be an option for “highly needed” infected implantable catheters in conjunction with systemic antibiotic therapy (BIII).	
• Empirical glycopeptide therapy is not recommended (DI).	
• Modify systemic antibiotic treatment according to microbiological results of susceptibility testing (AII).	
• Initial antimicrobial regimen may be continued in case of clinical response to empiric treatment without microbiological evidence of insufficient antibiotic coverage (BIII).	
• For uncomplicated CRI, continue antibiotic treatment ≥ 7 days depending on the causative pathogen, counting the day of the first sterile blood culture as day one of treatment (AII).	
• At least 2 weeks of systemic antimicrobial treatment is recommended in immunocompromised patients (BIII).	

*CRI*, catheter-related infection; *CRBSI*, catheter-related bloodstream infection; *CVC*, central venous catheter

**Table 6:** Antimicrobial therapy of CRI depending on causative pathogen

Pathogen	Therapy	Duration^a^
*Staphylococcus aureus* (methicillin-sensitive)^b^	Isoxazolyl penicillin (anti-staphylococcal penicillin)	≥ 2 weeks^c^
*Staphylococcus aureus* (methicillin-resistant)^b^	Glycopeptide, linezolid, daptomycin	≥ 2 weeks^c^
		4–6 weeks in case of complicated infection
Coagulase-negative staphylococci	According to susceptibility pattern; glycopeptides only in case of methicillin resistance	5–7 days after defervescence (in pts with persistent neutropenia)
Enterococci	Aminopenicillin; glycopeptide and aminoglycoside in case of ampicillin resistance; linezolid in case of vancomycin resistance	5–7 days after defervescence (in pts with persistent neutropenia)
*Stenotrophomonas* spp.	Co-trimoxazole	≥ 2 weeks
	According to susceptibility pattern in case of allergy (e.g., levofloxacin)	
*Pseudomonas* spp.	According to susceptibility pattern	≥ 2 weeks
*Candida albicans*^b^	Echinocandin according to susceptibility pattern or amphotericin B lipid-based formulations after stabilization step down to fluconazole	≥ 2 weeks
Non-*albicans Candida* spp.^b^	Echinocandin; step down to azole according to susceptibility pattern or amphotericin B lipid-based	≥ 2 weeks (after first sterile blood culture)
All other pathogens	According to susceptibility pattern	Not defined

^a^Follow-up blood cultures necessary after cessation of antibiotic/antifungal therapy in order to rule out persistence of infection (AII)

^b^Early CVC removal required (AII)

^c^Higher incidence of organ infection if treatment is continued for < 2 weeks (AII)

*CVC*, central venous catheter; *pts*, patients

#### Catheter removal

Antimicrobial therapy and removal of the CVC are crucial in the treatment of patients with suspected CRI. As retention of the CVC in patients with suspected CRI can result in treatment failure or recurrence of infection in spite of antibiotic therapy, CVC removal is encouraged in all patients with CRI whenever possible [1, 21, 38, 86, 190–192]. Although CVC removal and reinsertion may be burdensome for cancer patients, early CVC removal is particularly encouraged in patients with deteriorating clinical state, sepsis, or septic shock and in case of severe complications such as endocarditis, septic thrombosis, abscess formations, or osteomyelitis [193]. In addition, in patients with tunnel or pocket infection, CVC removal is usually required [1]. CVC exchange is often cumbersome and associated with significant risks in thrombocytopenic patients and may not always be feasible. However, retention of CVC has not been tested as a strategy in any randomized trial in patients with suspected CRI. Therefore, in severely thrombocytopenic patients with limited venous access, the risk of CVC reinsertion should be carefully weighed against the risk of patient deterioration and prolongation of the CRI.

Several studies in patients with *S. aureus* bacteremia indicate an increased risk for hematogenous complications, relapse of infection, and death of infection if the CVC is retained after detection of *S. aureus* [194, 195]. Retrospective analyses reported successful preservation of Hickman catheters in patients with *S. aureus* bacteremia in 18–60% of analyzed cases. However, these studies are likely to be biased due to selection of patients with successful salvage [196, 197]. Moreover, El Zakhem and colleagues recently analyzed 299 cancer patients with 304 episodes of *S. aureus* CLABSI and reported a higher rate of relapse in patients whose CVC was retained beyond 3 days compared with those whose CVC was removed or exchanged within the first 3 days from the onset of bacteremia [198]. Therefore, early CVC removal is recommended in patients with suspected *S. aureus* CRI.

Retrospective studies suggest that mucositis and the gastrointestinal tract rather than the CVC might be the cause for candidemia in a large proportion of cancer patients with candidemia [199, 200]. However, the diagnosis of CRI in patients with candidemia without CVC removal is challenging, since a DTTP of > 2 h is unreliable in excluding *Candida*-related CRBSI, and the cutoff DTTP for different *Candida* spp. is not established and may vary substantially [16–18]. In two prospective observational studies [201, 202] and a retrospective analysis of two prospective trials testing the efficacy of antifungal drugs [203], early CVC removal was not associated with any clinical benefit in patients with candidemia and CVC. However, these studies were not limited to cancer patients, included only a minority of neutropenic patients, and did not use stringent criteria for the definition of CRBSI. In contrast, Raad and colleagues retrospectively analyzed 404 cancer patients with candidemia and CVCs and found that CVC removal within 72 h after onset of candidemia improved the response to antifungal therapy in patients with *Candida*-related CRBSI [199]. Similarly, findings from retrospective studies and a prospective cohort study in cancer patients with candidemia and systematic reviews indicate a decreased mortality in patients with CVC removal [204–206]. Therefore, prompt CVC removal is recommended in cancer patients with candidemia and yeast-related fungemias (e.g., *Rhodotorula* spp.) other than caused by *Cryptococcus* spp. [207, 208].

In patients with CRBSI caused by Gram-negative bacteria, CVC retention resulted in higher risk of relapse of Gram-negative bacteremia [8]. Furthermore, early CVC removal was associated with lower mortality in a single-center retrospective study including 78 cases of Gram-negative CRBSI (43 definite and 35 probable), of which about one-third had cancer [209]. Another retrospective study on 300 cancer patients with Gram-negative bloodstream infections showed that CVC removal within 2 days of pathogen detection was associated with lower overall mortality in CRBSI patients (overall mortality rate at 3-month follow-up: 3% and 19%, *P* = 0.01, in patients with early and delayed CVC removal, respectively) [191]. Similarly, prompt CVC removal was found to be associated with a better response to antimicrobial therapy and lower risk of mortality in patients with CRBSI related to *Stenotrophomonas* spp. [209–211]. Therefore, prompt CVC removal (within 48–72 h) is recommended in case of CRBSI caused by Gram-negative bacteria.

A recent study on 184 CRBSI episodes caused by CoNS including 41% cancer patients found that withholding antimicrobial therapy in CoNS CRBSI following CVC removal was not associated with non-resolved CRIs or mortality [212].

In patients with implanted long-term catheters such as Hickman catheters and port catheters, two retrospective cohort studies analyzed the effect of CVC removal in patients with CoNS CRBSI. The investigators found no impact on mortality or the resolution of bacteremia, although a higher risk of recurrence of infection was detectable [86, 213]. Thus, in hemodynamically stable patients of urgent need for long-term venous access and limited options, long-term CVCs may be left in place under careful surveillance and with systemic antibacterial therapy. Similarly, in retrospective studies examining the effect of CVC removal in patients with *Corynebacterium jeikeium* causing BSI and CRI, retention of the CVC was not associated with higher mortality or recurrence of infection if systemic antibiotic treatment was administered [214, 215]. Accordingly, in patients with *C. jeikeium* CRI, CVC retention along with systemic antibiotic treatment may be acceptable in hemodynamically stable patients with tunneled CVC under careful surveillance.

In patients with CVC left in place after onset of symptoms, CVC removal is warranted in any case of clinical deterioration or continued positive blood cultures 72 h after initiation of therapy in spite of appropriate antimicrobial therapy [1].

CVC exchange over a guidewire may induce bacteremia and therefore cannot be recommended as alternative approach to CVC removal. CVC exchange over a guidewire and replacement with a minocycline/rifampin-coated CVC were shown to prevent biofilm formation [216] and appeared to be safe and improved response to systemic antimicrobial therapy in one matched retrospective cohort study in cancer patients with CRI [217]. This approach may be feasible in selected patients when the risk of reinsertion outweighs the persistence or relapse of CRI.

#### Antibiotic lock therapy

ALT is conducted by instillation of antibiotic solutions at high concentrations mostly in combination with anticoagulants such as heparin or EDTA (ethylenediaminetetraacetate) into a CVC lumen for several hours. ALT was studied in small randomized trials and retrospective studies as treatment in conjunction with systemic antibiotic therapy for patients with implantable CVCs showing efficacy in up to 100% [218–220]. However, the procedure of ALT is not standardized, and considerable variability between different protocols concerning the antibiotic solutions used, antibiotic concentrations, dwelling time, the duration of ALT, and the simultaneous use of the infected catheter has been reported [221]. Moreover, ALT may be less effective in CRBSI caused by *S. aureus*, *Candida* spp., and other microorganisms embedded in biofilms [222]. In conclusion, ALT may be a treatment option for patients with infected implantable CVCs and limited options for vascular access in conjunction with systemic antibiotic therapy.

Several antiseptic solutions such as ethanol or combinations of antibiotics with antiseptic solutions have been tested in smaller studies in patients with bacteremia [223–226]. In a randomized study using either ethanol 70% or saline in 94 children with cancer as treatment or secondary prophylaxis for CLABSI, ALT did not prevent CLABSI treatment failure and it increased CVC occlusion [224]. In contrast, approaches using minocycline-EDTA-ethanol solution while leaving the catheter in place compared favorably in 30 adult cancer patients with CLABSI in terms of duration of systemic antimicrobial therapy and mechanical complications. However, as this study only used a historic control of patients, the results should be confirmed in a randomized trial [227].

#### Systemic antimicrobial treatment

Systemic antibiotic treatment is the second mainstay of treatment of CRI and should be started immediately after sampling of blood cultures. The choice of empiric antibiotic treatment depends on the clinical severity of the infection, the patient’s comorbidities, and potential known colonization with MDR bacteria, as well as local resistance patterns [45, 87]. In high-risk neutropenic patients, piperacillin/tazobactam, imipenem, and meropenem can be suitable options for first-line empirical antibacterial therapy [87]. Empiric addition of glycopeptides prior to microbiological evidence of Gram-positive CRI is discouraged, as this treatment might result in an increase in antibiotic resistance and additional toxicity and does not improve outcomes of febrile neutropenic patients with cancer as shown in several studies and a recent meta-analysis [228].

Empiric treatment should be modified according to microbiological results of susceptibility testing. In patients clinically responding to empiric treatment without microbiological evidence of insufficient antibiotic coverage, the initial antimicrobial regimen may be continued. Repeated blood cultures are recommended to account for the first day of negative blood culture results to guide treatment duration and to detect complicated CRI with prolonged microbiological evidence of bacteremia in spite of antimicrobial treatment. Duration of the treatment depends on the pathogen detected, the resolution of symptoms, the absence or emergence of complications such as endocarditis or osteomyelitis, and clinical, microbiological, and laboratory evidence of response to antimicrobial treatment [45, 87].

Depending on the causative pathogen and for uncomplicated CRI, antibiotic treatment should be continued for at least 7 days, counting the day of the first sterile blood culture as day one of treatment and catheter removal [1, 229, 230]. Longer treatment duration may be indicated in case of complications such as endocarditis and for treatment of specific pathogens such as *S. aureus*, *Candida* spp. and other fungi [207], *Stenotrophomonas* spp., and others (Table 6). Optimal treatment duration in neutropenic cancer patients is currently unclear, and whether treatment should be continued until resolution of neutropenia remains controversial as specific data from high-quality studies in neutropenic patients are lacking [231]. With respect to specific clinical scenarios such as non-response to antimicrobial treatment or the management of sepsis, we refer to recent AGIHO guidelines [45, 46, 87].

## Conclusion

In this guideline, we summarize recommendations on definition, diagnosis, management, and prevention of CRI in cancer patients. This publication replaces the current version of our guideline and adds specific recommendations on cancer patients in addition to institutional and regulatory guidelines.

Diagnostic procedures for the detection of CRIs should be initiated upon clinical signs and symptoms of infection in patients with any type of indwelling CVC. In patients with suspected CRI, at least two pairs of blood cultures with adequate quantity of blood should be taken simultaneously from the CVC and a peripheral vein. Although the DTTP has been reported as a sensitive and specific diagnostic marker for CRBSI, recent studies suggest that the DTTP is inaccurate for the diagnosis of *S. aureus*– and *Candida* spp.–associated CRBSI. In case of suspected or diagnosed CRI, the mainstays of treatment are antimicrobial therapy and removal of the CVC. As CVC retention may result in treatment failure or recurrence of infection in spite of antibiotic therapy, removal is encouraged whenever possible. CVC retention along with systemic antibiotic treatment may be acceptable in hemodynamically stable patients under careful surveillance in certain cases and for selected pathogens. In any case, removal is warranted in case of clinical deterioration or continued positive blood cultures 72 h after initiation of appropriate antimicrobial treatment. Systemic antibiotic treatment should be initiated immediately after sampling of blood cultures and chosen depending on severity of the infection, patient’s comorbidities, and potential colonization with MDR pathogens as well as local resistance patterns.

## Data Availability

Not applicable.

## References

[1] Mermel LA, Allon M, Bouza E (2009). Clinical practice guidelines for the diagnosis and management of intravascular catheter-related infection: 2009 update by the Infectious Diseases Society of America. Clin Infect Dis.

[2] Raad I, Chaftari AM (2014). Advances in prevention and management of central line-associated bloodstream infections in patients with cancer. Clin Infect Dis.

[3] Gallieni M, Pittiruti M, Biffi R (2008). Vascular access in oncology patients. CA Cancer J Clin.

[4] Aghdassi SJS, Schröder C, Gruhl D (2019). Point prevalence survey of peripheral venous catheter usage in a large tertiary care university hospital in Germany. Antimicrob Resist Infect Control.

[5] Baier C, Linke L, Eder M (2020). Incidence, risk factors and healthcare costs of central line-associated nosocomial bloodstream infections in hematologic and oncologic patients. PLoS One.

[6] Rabensteiner J, Theiler G, Duettmann W (2015). Detection of central venous catheter-related bloodstream infections in haematooncological patients. Eur J Clin Investig.

[7] Hanna HA, Raad I (2001). Blood products: a significant risk factor for long-term catheter-related bloodstream infections in cancer patients. Infect Control Hosp Epidemiol.

[8] Hanna H, Afif C, Alakech B (2004). Central venous catheter–related bacteremia due to Gram-negative bacilli: significance of catheter removal in preventing relapse. Infect Control Hosp Epidemiol.

[9] Umscheid CA, Mitchell MD, Doshi JA (2011). Estimating the proportion of healthcare-associated infections that are reasonably preventable and the related mortality and costs. Infect Control Hosp Epidemiol.

[10] Hentrich M, Schalk E, Schmidt-Hieber M (2014). Central venous catheter-related infections in hematology and oncology: 2012 updated guidelines on diagnosis, management and prevention by the Infectious Diseases Working Party of the German Society of Hematology and Medical Oncology. Ann Oncol.

[11] Kish MA (2001). Guide to development of practice guidelines. Clin Infect Dis.

[12] Chatzinikolaou I, Hanna H, Hachem R (2004). Differential quantitative blood cultures for the diagnosis of catheter-related bloodstream infections associated with short- and long-term catheters: a prospective study. Diagn Microbiol Infect Dis.

[13] Safdar N, Fine JP, Maki DG (2005) Meta-analysis: methods for diagnosing intravascular device-related bloodstream infection. Ann Intern Med 10.7326/0003-4819-142-6-200503150-00011 15767623

[14] Kaasch AJ, Rieg S, Hellmich M et al (2014) Differential time to positivity is not predictive for central line-related Staphylococcus aureus bloodstream infection in routine clinical care. J Inf Secur. 10.1016/j.jinf.2013.08.006 10.1016/j.jinf.2013.08.006 23954613

[15] Bouzidi H, Emirian A, Marty A (2018). Differential time to positivity of central and peripheral blood cultures is inaccurate for the diagnosis of Staphylococcus aureus long-term catheter-related sepsis. J Hosp Infect.

[16] Park KH, Lee MS, Lee SO (2014). Diagnostic usefulness of differential time to positivity for catheter-related candidemia. J Clin Microbiol.

[17] Bouza E, Alcalá L, Muñoz P et al (2013) Can microbiologists help to assess catheter involvement in candidaemic patients before removal? Clin Microbiol Infect 19. 10.1111/1469-0691.12096 10.1111/1469-0691.12096 23231412

[18] Jo KM, Choi S, Jung KH (2020). Diagnostic usefulness of differential time to positivity in neutropenic cancer patients with suspected catheter-related candidemia. Med Mycol.

[19] Gits-Muselli M, Villiers S, Hamane S (2020). Time to and differential time to blood culture positivity for assessing catheter-related yeast fungaemia: a longitudinal, 7-year study in a single university hospital. Mycoses.

[20] See I, Iwamoto M, Allen-Bridson K (2013). Mucosal barrier injury laboratory-confirmed bloodstream infection: results from a field test of a new National Healthcare Safety Network definition. Infect Control Hosp Epidemiol.

[21] Zakhour R, Chaftari AM, Raad II (2016). Catheter-related infections in patients with haematological malignancies: novel preventive and therapeutic strategies. Lancet Infect Dis.

[22] Chaftari AM, Jordan M, Hachem R (2016). A clinical practical approach to the surveillance definition of central line–associated bloodstream infection in cancer patients with mucosal barrier injury. Am J Infect Control.

[23] Freeman JT, Elinder-Camburn A, McClymont C (2013). Central line–associated bloodstream infections in adult hematology patients with febrile neutropenia an evaluation of surveillance definitions using differential time to blood culture positivity. Infect Control Hosp Epidemiol.

[24] Metzger KE, Rucker Y, Callaghan M (2015). The burden of mucosal barrier injury laboratory-confirmed bloodstream infection among hematology, oncology, and stem cell transplant patients. Infect Control Hosp Epidemiol.

[25] O’Grady NP, Alexander M, Burns LA et al (2011) Guidelines for the prevention of intravascular catheter-related infections. Clin Infect Dis:52. 10.1093/cid/cir257 10.1093/cid/cir257 PMC3106269 21460264

[26] de Grooth HJ, Timsit JF, Mermel L (2020). Validity of surrogate endpoints assessing central venous catheter-related infection: evidence from individual- and study-level analyses. Clin Microbiol Infect.

[27] Robert K für K und I (KRINKO) beim, Koch-Institut R (2017) Prevention of vascular catheter-related infections: part 1-nontunneled central venous catheters: recommendation of the Commission for Hospital Hygiene and Infection Prevention (KRINKO) at the Robert Koch Institute

[28] Steinberg JP, Coffin SE (2013). Improving the central line—associated bloodstream infection surveillance definition: a work in progress. Infect Control Hosp Epidemiol.

[29] Epstein L, See I, Edwards JR (2016). Mucosal barrier injury laboratory-confirmed bloodstream infections (MBI-LCBI): descriptive analysis of data reported to national healthcare safety network (NHSN), 2013. Infect Control Hosp Epidemiol.

[30] Kato Y, Hagihara M, Kurumiya A (2018). Impact of mucosal barrier injury laboratory-confirmed bloodstream infection (MBI-LCBI) on central line-associated bloodstream infections (CLABSIs) in department of hematology at single university hospital in Japan. J Infect Chemother.

[31] Biehl LM, Huth A, Panse J (2016). A randomized trial on chlorhexidine dressings for the prevention of catheter-related bloodstream infections in neutropenic patients. Ann Oncol.

[32] Schalk E, Teschner D, Hentrich M (2019). Central venous catheter-related bloodstream infections in patients with hematological malignancies: comparison of data from a clinical registry and a randomized controlled trial. Infect Control Hosp Epidemiol.

[33] Schalk E, Biehl LM, Färber J (2017). Determination of a cutoff time point for prophylactic exchange of central venous catheters for prevention of central venous catheter–related bloodstream infections in patients with hematological malignancies. Infect Control Hosp Epidemiol.

[34] Schalk E, Vehreschild MJGT, Biehl LM (2020) Influence of different definitions of central venous catheter–related bloodstream infections on epidemiological parameters in cancer patients. 1–2. 10.1017/ice.2020.274 10.1017/ice.2020.274 32631475

[35] Linares J (2007). Diagnosis of catheter-related bloodstream infection: conservative techniques. Clin Infect Dis.

[36] Safdar N, Maki DG (2004). The pathogenesis of catheter-related bloodstream infection with noncuffed short-term central venous catheters. Intensive Care Med.

[37] Luft D, Schmoor C, Wilson C (2010). Central venous catheter-associated bloodstream infection and colonisation of insertion site and catheter tip. What are the rates and risk factors in haematology patients?. Ann Hematol.

[38] Raad I (1998). Intravascular-catheter-related infections. Lancet.

[39] Dube WC, Jacob JT, Zheng Z (2020). Comparison of rates of central line-associated bloodstream infections in patients with 1 vs 2 central venous catheters. JAMA Netw Open.

[40] Peris A, Zagli G, Bonizzoli M (2010). Implantation of 3951 long-term central venous catheters: performances, risk analysis, and patient comfort after ultrasound-guidance introduction. Anesth Analg.

[41] Mollee P, Jones M, Stackelroth J (2011). Catheter-associated bloodstream infection incidence and risk factors in adults with cancer: a prospective cohort study. J Hosp Infect.

[42] Touré A, Vanhems P, Lombard-Bohas C (2012). Totally implantable central venous access port infections in patients with digestive cancer: incidence and risk factors. Am J Infect Control.

[43] Howell PB, Walters PE, Donowitz GR, Farr BM (1995). Risk factors for infection of adult patients with cancer who have tunnelled central venous catheters. Cancer.

[44] Wisplinghoff H, Seifert H, Wenzel RP, Edmond MB (2003). Current trends in the epidemiology of nosocomial bloodstream infections in patients with hematological malignancies and solid neoplasms in hospitals in the United States. Clin Infect Dis.

[45] Kochanek M, Schalk E, von Bergwelt-Baildon M (2019). Management of sepsis in neutropenic cancer patients: 2018 guidelines from the Infectious Diseases Working Party (AGIHO) and Intensive Care Working Party (iCHOP) of the German Society of Hematology and Medical Oncology (DGHO). Ann Hematol.

[46] Kiehl MG, Beutel G, Böll B (2018). Consensus statement for cancer patients requiring intensive care support. Ann Hematol.

[47] Tölle D, Hentrich M, Pelzer BW (2019). Impact of neutropenia on central venous catheter-related bloodstream infections in patients with hematological malignancies at the time of central venous catheter insertion: a matched-pair analysis. Infect Control Hosp Epidemiol.

[48] McDonald MK, Culos KA, Gatwood KS (2018). Defining incidence and risk factors for catheter-associated bloodstream infections in an outpatient adult hematopoietic cell transplantation program. Biol Blood Marrow Transplant.

[49] Richters A, Van Vliet M, Peer PGM (2014). Incidence of and risk factors for persistent gram-positive bacteraemia and catheter-related thrombosis in haematopoietic stem cell transplantation. Bone Marrow Transplant.

[50] Lordick F, Hentrich M, Decker T (2003). Ultrasound screening for internal jugular vein thrombosis aids the detection of central venous catheter-related infections in patients with haemato-oncological diseases: a prospective observational study. Br J Haematol.

[51] Raad II, Luna M, Khalil SAM (1994). The relationship between the thrombotic and infectious complications of central venous catheters. JAMA J Am Med Assoc.

[52] Meyer E, Beyersmann J, Bertz H (2007). Risk factor analysis of blood stream infection and pneumonia in neutropenic patients after peripheral blood stem-cell transplantation. Bone Marrow Transplant.

[53] Lipitz-Snyderman A, Sepkowitz KA, Elkin EB et al (2014) Long-term central venous catheter use and risk of infection in older adults with cancer. J Clin Oncol. 10.1200/JCO.2013.53.3018 10.1200/JCO.2013.53.3018 PMC4105488 24982458

[54] Fang S, Yang J, Song L (2017). Comparison of three types of central venous catheters in patients with malignant tumor receiving chemotherapy. Patient Prefer Adherence.

[55] Groeger JS, Lucas AB, Thaler HT (1993). Infectious morbidity associated with long-term use of venous access devices in patients with cancer. Ann Intern Med.

[56] Patel GS, Jain K, Kumar R (2014). Comparison of peripherally inserted central venous catheters (PICC) versus subcutaneously implanted port-chamber catheters by complication and cost for patients receiving chemotherapy for non-haematological malignancies. Support Care Cancer.

[57] Cotogni P, Barbero C, Garrino C (2015). Peripherally inserted central catheters in non-hospitalized cancer patients: 5-year results of a prospective study. Support Care Cancer.

[58] Chopra V, O’Horo JC, Rogers MAM (2013). The risk of bloodstream infection associated with peripherally inserted central catheters compared with central venous catheters in adults: a systematic review and meta-analysis. Infect Control Hosp Epidemiol.

[59] Bellesi S, Chiusolo P, De Pascale G (2013). Peripherally inserted central catheters (PICCs) in the management of oncohematological patients submitted to autologous stem cell transplantation. Support Care Cancer.

[60] Kang J, Chen W, Sun W (2017). Peripherally inserted central catheter-related complications in cancer patients: a prospective study of over 50,000 catheter days. J Vasc Access.

[61] Lee JH, Kim ET, Shim DJ (2019). Prevalence and predictors of peripherally inserted central catheter-associated bloodstream infections in adults: a multicenter cohort study. PLoS One.

[62] Campagna S, Gonella S, Berchialla P et al (2019) Can peripherally inserted central catheters be safely placed in patients with cancer receiving chemotherapy? A retrospective study of almost 400,000 catheter-days. Oncologist:24. 10.1634/theoncologist.2018-0281 10.1634/theoncologist.2018-0281 PMC6738314 30755503

[63] Mariggiò E, Iori AP, Micozzi A et al (2020) Peripherally inserted central catheters in allogeneic hematopoietic stem cell transplant recipients. Support Care Cancer. 10.1007/s00520-019-05269-z 10.1007/s00520-019-05269-z 31900609

[64] McBride KD, Fisher R, Warnock N (1997). A comparative analysis of radiological and surgical placement of central venous catheters. Cardiovasc Intervent Radiol.

[65] Tomlinson D, Mermel LA, Ethier MC (2011). Defining bloodstream infections related to central venous catheters in patients with cancer: a systematic review. Clin Infect Dis.

[66] Sotir MJ, Lewis C, Bisher EW (1999). Epidemiology of device-associated infections related to a long-term implantable vascular access device. Infect Control Hosp Epidemiol.

[67] Worth LJ, Black J, Seymour JF (2008). Surveillance for catheter-associated bloodstream infection in hematology units: quantifying the characteristics of a practical case definition. Infect Control Hosp Epidemiol.

[68] DiGiorgio MJ, Fatica C, Oden M (2012). Development of a modified surveillance definition of central line–associated bloodstream infections for patients with hematologic malignancies. Infect Control Hosp Epidemiol.

[69] Dettenkofer M, Wilson C, Gratwohl A (2010). Skin disinfection with octenidine dihydrochloride for central venous catheter site care: a double-blind, randomized, controlled trial. Clin Microbiol Infect.

[70] Gudiol C, Garcia-Vidal C, Arnan M (2014). Etiology, clinical features and outcomes of pre-engraftment and post-engraftment bloodstream infection in hematopoietic SCT recipients. Bone Marrow Transplant.

[71] Chaberny IF, Ruseva E, Sohr D (2009). Surveillance with successful reduction of central line-associated bloodstream infections among neutropenic patients with hematologic or oncologic malignancies. Ann Hematol.

[72] Dettenkofer M, Wenzler-Rottele S, Babikir R (2005). Surveillance of nosocomial sepsis and pneumonia in patients with a bone marrow or peripheral blood stem cell transplant: a multicenter project. Clin Infect Dis.

[73] German Reference Center for Surveillance of Nosocomial Infections. ONKO-KISS Module

[74] Seifert H, Cornely O (2003) Cancer patients related to short-term nontunnelled catheters determined by quantitative blood cultures, differential time to positivity, and molecular epidemiological. J Clin Densitom … 41:118–123. 10.1128/JCM.41.1.118 10.1128/JCM.41.1.118-123.2003 PMC149641 12517836

[75] Hummel M, Warga C, Hof H (2009). Diagnostic yield of blood cultures from antibiotic-nave and antibiotically treated patients with haematological malignancies and high-risk neutropenia. Scand J Infect Dis.

[76] Wisplinghoff H, Bischoff T, Tallent SM, et al (2004) Cases from a prospective nationwide surveillance study. BSI US Hosp • CID 309:179 10.1086/421946 15306996

[77] Marcos M, Soriano A, Iñurrieta A (2011). Changing epidemiology of central venous catheter-related bloodstream infections: increasing prevalence of Gram-negative pathogens. J Antimicrob Chemother.

[78] Braun E, Hussein K, Geffen Y (2014). Predominance of Gram-negative bacilli among patients with catheter-related bloodstream infections. Clin Microbiol Infect.

[79] Chaftari AM, Hachem R, Jiang Y (2018). Changing epidemiology of catheter-related bloodstream infections in cancer patients. Infect Control Hosp Epidemiol.

[80] Dutcher K, Lederman ER, Brodine S, Patel S (2013). Impact of the 2013 revised Centers for Disease Control and Prevention central line–associated bloodstream infection (CLABSI) surveillance definition on inpatient hospital CLABSI rates: is it enough?. Infect Control Hosp Epidemiol.

[81] Schmidt-Hieber M, Bierwirth J, Buchheidt D (2018). Diagnosis and management of gastrointestinal complications in adult cancer patients: 2017 updated evidence-based guidelines of the Infectious Diseases Working Party (AGIHO) of the German Society of Hematology and Medical Oncology (DGHO). Ann Hematol.

[82] Gudiol C, Bodro M, Simonetti A (2013). Changing aetiology, clinical features, antimicrobial resistance, and outcomes of bloodstream infection in neutropenic cancer patients. Clin Microbiol Infect.

[83] Gustinetti G, Mikulska M (2016). Bloodstream infections in neutropenic cancer patients: a practical update. Virulence.

[84] Montassier E, Batard E, Gastinne T (2013). Recent changes in bacteremia in patients with cancer: a systematic review of epidemiology and antibiotic resistance. Eur J Clin Microbiol Infect Dis.

[85] Cairo J, Hachem R, Rangaraj G (2011). Predictors of catheter-related gram-negative bacilli bacteraemia among cancer patients. Clin Microbiol Infect.

[86] Coyle VM, McMullan R, Morris TCM (2004). Catheter-related bloodstream infection in adult haematology patients: catheter removal practice and outcome. J Hosp Infect.

[87] Heinz WJ, Buchheidt D, Christopeit M (2017). Diagnosis and empirical treatment of fever of unknown origin (FUO) in adult neutropenic patients: guidelines of the Infectious Diseases Working Party (AGIHO) of the German Society of Hematology and Medical Oncology (DGHO). Ann Hematol.

[88] Bouza E, Alvarado N, Alcala L (2007). A randomized and prospective study of 3 procedures for the diagnosis of catheter-related bloodstream infection without catheter withdrawal. Clin Infect Dis.

[89] DesJardin JA, Falagas ME, Ruthazer R (1999). Clinical utility of blood cultures drawn from indwelling central venous catheters in hospitalized patients with cancer. Ann Intern Med.

[90] Guembe M, Rodríguez-Créixems M, Sánchez-Carrillo C (2012). Differential time to positivity (DTTP) for the diagnosis of catheter-related bloodstream infection: do we need to obtain one or more peripheral vein blood cultures?. Eur J Clin Microbiol Infect Dis.

[91] Dobbins BM, Catton JA, Kite P (2003). Each lumen is a potential source of central venous catheter-related bloodstream infection. Crit Care Med.

[92] Catton JA, Dobbins BM, Kite P (2005). In situ diagnosis of intravascular catheter-related bloodstream infection: a comparison of quantitative culture, differential time to positivity, and endoluminal brushing. Crit Care Med.

[93] Krause R, Valentin T, Salzer H (2013). Which lumen is the source of catheter-related bloodstream infection in patients with multi-lumen central venous catheters?. Infection.

[94] Planes AM, Calleja R, Bernet A (2016). Evaluación de la utilidad del hemocultivo cuantitativo en el diagnóstico de la bacteriemia relacionada con catéter: análisis comparativo de dos periodos (2002 y 2012). Enferm Infecc Microbiol Clin.

[95] Blot F, Nitenberg GE, Chachaty E (1999). Diagnosis of catheter-related bacteraemia: a prospective comparison of the time to positivity of hub-blood versus peripheral-blood cultures. Lancet.

[96] Raad I, Hanna HA, Alakech B et al (2004) Differential time to positivity: a useful method for diagnosing catheter-related bloodstream infections. Ann Intern Med. 10.7326/0003-4819-140-1-200401060-00007 10.7326/0003-4819-140-1-200401060-00007 14706968

[97] Abdelkefi A, Achour W, Ben Othman T (2005). Difference in time to positivity is useful for the diagnosis of catheter-related bloodstream infection in hematopoietic stem cell transplant recipients. Bone Marrow Transplant.

[98] Rijnders BJA, Van Wijngaerden E, Peetermans WE (2002). Catheter-tip colonization as a surrogate end point in clinical studies on catheter-related bloodstream infection: how strong is the evidence?. Clin Infect Dis.

[99] Rijnders BJA (2007). Diagnosing catheter-related bloodstream infection without catheter removal? Not so fast!. Clin Infect Dis.

[100] Guembe M, Martín-Rabadán P, Echenagusia A (2013). Value of superficial cultures for prediction of catheter-related bloodstream infection in long-term catheters: a prospective study. J Clin Microbiol.

[101] Eggimann P, Harbarth S, Constantin MN (2000). Impact of a prevention strategy targeted at vascular-access care on incidence of infections acquired in intensive care. Lancet.

[102] Pronovost P, Needham D, Berenholtz S et al (2006) An intervention to decrease catheter-related bloodstream infections in the ICU. N Engl J Med. 10.1056/NEJMoa061115 10.1056/NEJMoa061115 17192537

[103] Pronovost PJ, Goeschel CA, Colantuoni E (2010). Sustaining reductions in catheter related bloodstream infections in Michigan intensive care units: observational study. BMJ.

[104] Guerin K, Wagner J, Rains K, Bessesen M (2010). Reduction in central line-associated bloodstream infections by implementation of a postinsertion care bundle. Am J Infect Control.

[105] Peredo R, Sabatier C, Villagrá A (2010). Reduction in catheter-related bloodstream infections in critically ill patients through a multiple system intervention. Eur J Clin Microbiol Infect Dis.

[106] Ista E, van der Hoven B, Kornelisse RF (2016). Effectiveness of insertion and maintenance bundles to prevent central-line-associated bloodstream infections in critically ill patients of all ages: a systematic review and meta-analysis. Lancet Infect Dis.

[107] Blot K, Bergs J, Vogelaers D (2014). Prevention of central line-associated bloodstream infections through quality improvement interventions: a systematic review and meta-analysis. Clin Infect Dis.

[108] Hugonnet S, Harbarth S, Sax H (2004). Nursing resources: a major determinant of nosocomial infection?. Curr Opin Infect Dis.

[109] Needleman J, Buerhaus P, Mattke S (2002). Nurse-staffing levels and the quality of care in hospitals. N Engl J Med.

[110] Chaftari P, Chaftari AM, Adachi J (2017). Improvement in the diagnosis of catheter-related bloodstream infections in a tertiary cancer center. Am J Infect Control.

[111] Higuera F, Rosenthal VD, Duarte P (2005). The effect of process control on the incidence of central venous catheter-associated bloodstream infections and mortality in intensive care units in Mexico. Crit Care Med.

[112] Choi SW, Chang L, Hanauer DA (2013). Rapid reduction of central line infections in hospitalized pediatric oncology patients through simple quality improvement methods. Pediatr Blood Cancer.

[113] Ibrahim KY, Pierrotti LC, Freire MP (2013). Health care-associated infections in hematology-oncology patients with neutropenia: a method of surveillance. Am J Infect Control.

[114] Colombier MA, Lafaurie M, de Fontbrune FS (2016). Usefulness of daily surveillance blood cultures in allogeneic hematopoietic stem cell transplant recipients on steroids: a 1-year prospective study. Transpl Infect Dis.

[115] Ghazal SS, Stevens MP, Bearman GM, Edmond MB (2014). Utility of surveillance blood cultures in patients undergoing hematopoietic stem cell transplantation. Antimicrob Resist Infect Control.

[116] Kameda K, Kimura S, Akahoshi Y (2016). High incidence of afebrile bloodstream infection detected by surveillance blood culture in patients on corticosteroid therapy after allogeneic hematopoietic stem cell transplantation. Biol Blood Marrow Transplant.

[117] Stohs E, Chow VA, Liu C (2019). Limited utility of outpatient surveillance blood cultures in hematopoietic cell transplant recipients on high-dose steroids for treatment of acute graft-versus-host-disease. Biol Blood Marrow Transplant.

[118] Raad II, Hohn DC, Gilbreath BJ et al (1994) Prevention of central venous catheter-related infections by using maximal sterile barrier precautions during insertion. Infect Control Hosp Epidemiol. 10.2307/30145574 8207189

[119] Cavanna L, Citterio C, Di Nunzio C (2020). Central venous catheterization in cancer patients with severe thrombocytopenia: ultrasound-guide improves safety avoiding prophylactic platelet transfusion. Mol Clin Oncol.

[120] Brass P, Hellmich M, Kolodziej L et al (2015) Ultrasound guidance versus anatomical landmarks for internal jugular vein catheterization SUMMARY OF FINDINGS FOR THE MAIN COMPARISON. Cochrane Database Syst Rev:CD006962. 10.1002/14651858.CD006962.pub2.www.cochranelibrary.com 10.1002/14651858.CD006962.pub2 PMC6517109 25575244

[121] Brass P, Hellmich M, Kolodziej L et al (2015) Ultrasound guidance versus anatomical landmarks for subclavian or femoral vein catheterization SO-: Cochrane Database of Systematic Reviews YR-: 2015 NO-: 1. Cochrane Database Syst Rev. 10.1002/14651858.CD011447.www.cochranelibrary.com 10.1002/14651858.CD011447 PMC6516998 25575245

[122] Imataki O, Shimatani M, Ohue Y, Uemura M (2019). Effect of ultrasound-guided central venous catheter insertion on the incidence of catheter-related bloodstream infections and mechanical complications. BMC Infect Dis.

[123] Hewlett AL, Rupp ME (2012) New developments in the prevention of intravascular catheter associated infections. Infect Dis Clin N Am 10.1016/j.idc.2011.09.002 22284372

[124] Maki DG, Alvarado CJ, Ringer M (1991) Prospective randomised trial of povidone-iodine, alcohol, and chlorhexidine for prevention of infection associated with central venous and arterial catheters. Lancet. 10.1016/0140-6736(91)90479-9 10.1016/0140-6736(91)90479-9 1677698

[125] Raad I, Hanna H, Maki D (2007). Intravascular catheter-related infections: advances in diagnosis, prevention, and management. Lancet Infect Dis.

[126] Lai NM, Lai NA, O’Riordan E et al (2016) Skin antisepsis for reducing central venous catheter-related infections. Cochrane Database Syst Rev 10.1002/14651858.CD010140.pub2 PMC6457952 27410189

[127] Hainsworth JD, Rubin MS, Spigel DR (2013). Molecular gene expression profiling to predict the tissue of origin and direct site-specific therapy in patients with carcinoma of unknown primary site: a prospective trial of the Sarah Cannon Research Institute. J Clin Oncol.

[128] Chaiyakunapruk N, Veenstra DL, Lipsky BA, Saint S (2002) Chlorhexidine compared with povidone-iodine solution for vascular catheter-site care: a meta-analysis. Ann Intern Med. 10.7326/0003-4819-136-11-200206040-00007 10.7326/0003-4819-136-11-200206040-00007 12044127

[129] Girard R, Comby C, Jacques D (2012). Alcoholic povidone-iodine or chlorhexidine-based antiseptic for the prevention of central venous catheter-related infections: in-use comparison. J Infect Public Health.

[130] Mimoz O, Lucet J-C, Kerforne T (2015). Chlorhexidine-alcohol versus povidone iodine-alcohol antisepsis for catheter-related infection prevention: an open-label, multicentre, randomised controlled trial. Intensive Care Med Exp.

[131] Ohtake S, Takahashi H, Nakagawa M (2018). One percent chlorhexidine-alcohol for preventing central venous catheter-related infection during intensive chemotherapy for patients with haematologic malignancies. J Infect Chemother.

[132] Mermel LA (2020). Sequential use of povidone-iodine and chlorhexidine for cutaneous antisepsis: a systematic review. Infect Control Hosp Epidemiol.

[133] Tietz A, Frei R, Dangel M et al (2005) Octenidine hydrochloride for the care of central venous catheter insertion sites in severely immunocompromised patients. Infect Control Hosp Epidemiol. 10.1086/502606 10.1086/502606 16156327

[134] Langgartner J, Linde HJ, Lehn N (2004). Combined skin disinfection with chlorhexidine/propanol and aqueous povidone-iodine reduces bacterial colonisation of central venous catheters. Intensive Care Med.

[135] Climo MW, Yokoe DS, Warren DK (2013). Effect of daily chlorhexidine bathing on hospital-acquired infection. N Engl J Med.

[136] Frost SA, Alogso MC, Metcalfe L (2016). Chlorhexidine bathing and health care-associated infections among adult intensive care patients: a systematic review and meta-analysis. Crit Care.

[137] Cobb DK, High KP, Sawyer RG et al (1992) A controlled trial of scheduled replacement of central venous and pulmonary-artery catheters. N Engl J Med. 10.1056/NEJM199210083271505 10.1056/NEJM199210083271505 1522842

[138] Cook D, Randolph A, Kernerman P et al (1997) Central venous catheter replacement strategies: a systematic review of the literature. Crit Care Med. 10.1097/00003246-199708000-00033 10.1097/00003246-199708000-00033 9267959

[139] Ullman AJ, Cooke ML, Gillies D et al (2013) Optimal timing for intravascular administration set replacement. Cochrane Database Syst Rev 2013. 10.1002/14651858.CD003588.pub3 10.1002/14651858.CD003588.pub3 PMC6516986 24037784

[140] Rasero L, Degl’Innocenti M, Mocali M et al (2000) Comparison of two different time interval protocols for central venous catheter dressing in bone marrow transplant patients: results of a randomized, multicenter study. Haematologica 10702816

[141] Ullman AJ, Cooke ML, Mitchell M et al (2015) Dressings and securement devices for central venous catheters (CVC). Cochrane Database Syst Rev 10.1002/14651858.CD010367.pub2 PMC6457749 26358142

[142] Dang FP, Li HJ, Tian JH (2019). Comparative efficacy of 13 antimicrobial dressings and different securement devices in reducing catheter-related bloodstream infections: a Bayesian network meta-analysis. Medicine (Baltimore).

[143] de Campos Pereira Silveira RC, dos Reis PED, Ferreira EB (2020). Dressings for the central venous catheter to prevent infection in patients undergoing hematopoietic stem cell transplantation: a systematic review and meta-analysis. Support Care Cancer.

[144] Ruschulte H, Franke M, Gastmeier P (2009). Prevention of central venous catheter related infections with chlorhexidine gluconate impregnated wound dressings: a randomized controlled trial. Ann Hematol.

[145] Timsit JF, Schwebel C, Bouadma L et al (2009) Chlorhexidine-impregnated sponges and less frequent dressing changes for prevention of catheter-related infections in critically III adults: a randomized controlled trial. JAMA - J Am Med Assoc. 10.1001/jama.2009.376 10.1001/jama.2009.376 19318651

[146] Timsit JF, Mimoz O, Mourvillier B (2012). Randomized controlled trial of chlorhexidine dressing and highly adhesive dressing for preventing catheter-related infections in critically ill adults. Am J Respir Crit Care Med.

[147] Eggimann P, Pagani JL, Dupuis-Lozeron E (2019). Sustained reduction of catheter-associated bloodstream infections with enhancement of catheter bundle by chlorhexidine dressings over 11 years. Intensive Care Med.

[148] Wei L, Li Y, Li X (2019). Chlorhexidine-impregnated dressing for the prophylaxis of central venous catheter-related complications: a systematic review and meta-analysis. BMC Infect Dis.

[149] Kamboj M, Blair R, Bell N (2015). Use of disinfection cap to reduce central-line—associated bloodstream infection and blood culture contamination among hematology—oncology patients. Infect Control Hosp Epidemiol.

[150] Patel PA, Boehm S, Zhou Y (2017). Prospective observational study on central line–associated bloodstream infections and central venous catheter occlusions using a negative displacement connector with an alcohol disinfecting cap. Am J Infect Control.

[151] Farkas JC, Liu N, Bleriot JP (1992). Single- versus triple-lumen central catheter-related sepsis: a prospective randomized study in a critically ill population. Am J Med.

[152] Ma TY, Yoshinaka R, Banaag A (1998). Total parenteral nutrition via multilumen catheters does not increase the risk of catheter-related sepsis: a randomized, prospective study. Clin Infect Dis.

[153] Dezfulian C, Lavelle J, Nallamothu BK (2003). Rates of infection for single-lumen versus multilumen central venous catheters: a meta-analysis. Crit Care Med.

[154] Yamamoto AJ, Solomon JA, Soulen MC (2002). Sutureless securement device reduces complications of peripherally inserted central venous catheters. J Vasc Interv Radiol.

[155] Goetz AM, Wagener MM, Miller JM, Muder RR (1998). Risk of infection due to central venous catheters: effect of site of placement and catheter type. Infect Control Hosp Epidemiol.

[156] Merrer J, De Jonghe B, Golliot F et al (2001) Complications of femoral and subclavian venous catheterization in critically III patients: a randomized controlled trial. J Am Med Assoc. 10.1001/jama.286.6.700 10.1001/jama.286.6.700 11495620

[157] Ge X, Cavallazzi R, Li C et al (2018) Central venous access sites for the prevention of venous thrombosis, stenosis and infection. Cochrane Database Syst Rev. 10.1002/14651858.CD004084.pub3 10.1002/14651858.CD004084.pub3 PMC6516884 22419292

[158] Mer M, Duse AG, Galpin JS, Richards GA (2009) Central venous catheterization: a prospective, randomized, double-blind study. Clin Appl Thromb. 10.1177/1076029608319878 10.1177/1076029608319878 18593746

[159] Parienti JJ, Mongardon N, Mégarbane B (2015). Intravascular complications of central venous catheterization by insertion site. N Engl J Med.

[160] Arvaniti K, Lathyris D, Blot S (2017). Cumulative evidence of randomized controlled and observational studies on catheter-related infection risk of central venous catheter insertion site in ICU patients: a pairwise and network meta-analysis. Crit Care Med.

[161] Parienti JJ (2017). Catheter-related bloodstream infection in jugular versus subclavian central catheterization. Crit Care Med.

[162] Heidenreich D, Hansen E, Kreil S et al (2020) Influence of the insertion site on central venous catheter-related complications in patients undergoing allogeneic hematopoietic cell transplantation. Biol Blood Marrow Transplant. 10.1016/j.bbmt.2020.02.007 10.1016/j.bbmt.2020.02.007 32084541

[163] Lennon M, Zaw NN, Pöpping DM, Wenk M (2012). Procedural complications of central venous catheter insertion. Minerva Anestesiol.

[164] Björkander M, Bentzer P, Schött U (2019). Mechanical complications of central venous catheter insertions: a retrospective multicenter study of incidence and risks. Acta Anaesthesiol Scand.

[165] Veenstra DL, Saint S, Saha S et al (1999) Efficacy of antiseptic-impregnated central venous catheters in preventing catheter-related bloodstream infection: a meta-analysis. J Am Med Assoc 10.1001/jama.281.3.261 9918482

[166] Ostendorf T, Meinhold A, Harter C (2005). Chlorhexidine and silver-sulfadiazine coated central venous catheters in haematological patients-a double-blind, randomised, prospective, controlled trial. Support Care Cancer.

[167] Chong HY, Lai NM, Apisarnthanarak A, Chaiyakunapruk N (2017). Comparative efficacy of antimicrobial central venous catheters in reducing catheter-related bloodstream infections in adults: abridged Cochrane systematic review and network meta-analysis. Clin Infect Dis.

[168] Lai NM, Chaiyakunapruk N, Lai NA et al (2016) Catheter impregnation, coating or bonding for reducing central venous catheter-related infections in adults. Cochrane Database Syst Rev 2016. 10.1002/14651858.CD007878.pub3 10.1002/14651858.CD007878.pub3 PMC6517176 26982376

[169] Jaeger K, Zenz S, Jüttner B (2005). Reduction of catheter-related infections in neutropenic patients: a prospective controlled randomized trial using a chlorhexidine and silver sulfadiazine-impregnated central venous catheter. Ann Hematol.

[170] Raad I, Darouiche R, Dupuis J (1997). Central venous catheters coated with minocycline and rifampin for the prevention of catheter-related colonization and bloodstream infections: a randomized, double-blind trial. Ann Intern Med.

[171] Darouiche RO, Raad II, Heard SO et al (1999) A comparison of two antimicrobial-impregnated central venous catheters. N Engl J Med. 10.1056/NEJM199901073400101 10.1056/NEJM199901073400101 9878638

[172] Darouiche RO, Berger DH, Khardori N (2005). Comparison of antimicrobial impregnation with tunneling of long-term central venous catheters: a randomized controlled trial. Ann Surg.

[173] Hanna H, Benjamin R, Chatzinikolaou I (2004). Long-term silicone central venous catheters impregnated with minocycline and rifampin decrease rates of catheter-related bloodstream infection in cancer patients: a prospective randomized clinical trial. J Clin Oncol.

[174] Yücel N, Lefering R, Maegele M (2004). Reduced colonization and infection with miconazole-rifampicin modified central venous catheters: a randomized controlled clinical trial. J Antimicrob Chemother.

[175] León C, Ruiz-Santana S, Rello J (2004). Benefits of minocycline and rifampin-impregnated central venous catheters: a prospective, randomized, double-blind, controlled, multicenter trial. Intensive Care Med.

[176] Bonne S, Mazuski JE, Sona C (2015). Effectiveness of minocycline and rifampin vs chlorhexidine and silver sulfadiazine-impregnated central venous catheters in preventing central line-associated bloodstream infection in a high-volume academic intensive care unit: a before and after trial. J Am Coll Surg.

[177] Chemaly RF, Sharma PS, Youssef S (2010). The efficacy of catheters coated with minocycline and rifampin in the prevention of catheter-related bacteremia in cancer patients receiving high-dose interleukin-2. Int J Infect Dis.

[178] van de Wetering MD, van Woensel JBM, Lawrie TA (2016) Prophylactic antibiotics for preventing Gram positive infections associated with long-term central venous catheters in oncology patients (review). 10.1002/14651858.CD003295.pub3.www.cochranelibrary.com 10.1002/14651858.CD003295.pub3 PMC6457614 24277633

[179] Ziegler M, Landsburg D, Pegues D (2019). Fluoroquinolone prophylaxis is highly effective for the prevention of central line–associated bloodstream infections in autologous stem cell transplant patients. Biol Blood Marrow Transplant.

[180] Zakrzewska-Bode A, Muytjens HL, Liem KD, Hoogkamp-Korstanje JAA (1995). Mupirocin resistance in coagulase-negative staphylococci, after topical prophylaxis for the reduction of colonization of central venous catheters. J Hosp Infect.

[181] Goossens GA, Jérôme M, Janssens C (2013). Comparing normal saline versus diluted heparin to lock non-valved totally implantable venous access devices in cancer patients: a randomised, non-inferiority, open trial. Ann Oncol.

[182] Gudiol C, Arnan M, Aguilar-Guisado M et al (2020) A randomized, double-blind, placebo-controlled trial (TAURCAT study) of citrate lock solution for prevention of endoluminal central venous catheter infection in neutropenic hematological patients. Antimicrob Agents Chemother. 10.1128/AAC.01521-19 10.1128/AAC.01521-19 PMC6985755 31712211

[183] Sanders J, Pithie A, Ganly P (2008). A prospective double-blind randomized trial comparing intraluminal ethanol with heparinized saline for the prevention of catheter-associated bloodstream infection in immunosuppressed haematology patients. J Antimicrob Chemother.

[184] Schoot RA, Van Ommen CH, Stijnen T (2015). Prevention of central venous catheter-associated bloodstream infections in paediatric oncology patients using 70% ethanol locks: a randomised controlled multi-centre trial. Eur J Cancer.

[185] Slobbe L, Doorduijn JK, Lugtenburg PJ (2010). Prevention of catheter-related bacteremia with a daily ethanol lock in patients with tunnelled catheters: a randomized, placebo-controlled trial. PLoS One.

[186] Worth LJ, Slavin MA, Heath S (2014). Ethanol versus heparin locks for the prevention of central venous catheter-associated bloodstream infections: a randomized trial in adult haematology patients with Hickman devices. J Hosp Infect.

[187] Carratalà J, Niubó J, Fernández-Sevilla A (1999). Randomized, double-blind trial of an antibiotic-lock technique for prevention of gram-positive central venous catheter-related infection in neutropenic patients with cancer. Antimicrob Agents Chemother.

[188] Safdar N, Maki DG (2006). Use of vancomycin-containing lock or flush solutions for prevention of bloodstream infection associated with central venous access devices: a meta-analysis of prospective, randomized trials. Clin Infect Dis.

[189] Zacharioudakis IM, Zervou FN, Arvanitis M (2014). Antimicrobial lock solutions as a method to prevent central line-associated bloodstream infections: a meta-analysis of randomized controlled trials. Clin Infect Dis.

[190] Bassetti M, Merelli M, Ansaldi F (2015). Clinical and therapeutic aspects of candidemia: a five year single centre study. PLoS One.

[191] Fares J, Khalil M, Chaftari AM (2019). Impact of catheter management on clinical outcome in adult cancer patients with Gram-negative bacteremia. Open Forum Infect Dis.

[192] Kim EJ, Kim YC, Ahn JY (2019). Risk factors for mortality in patients with Stenotrophomonas maltophilia bacteremia and clinical impact of quinolone-resistant strains. BMC Infect Dis.

[193] Raad S, Chaftari AM, Hachem RY (2018). Removal and insertion of central venous catheters in cancer patients is associated with high symptom burden. Expert Rev Med Devices.

[194] Fowler VG, Sanders LL, Sexton DJ (1998). Outcome of Staphylococcus aureus bacteremia according to compliance with recommendations of infectious diseases specialists: experience with 244 patients. Clin Infect Dis.

[195] Fowler VG, Justice A, Moore C (2005). Risk factors for hematogenous complications of intravascular catheter-associated Staphylococcus aureus bacteremia. Clin Infect Dis.

[196] Dugdale DC, Ramsey PG (1990). Staphylococcus aureus bacteremia in patients with Hickman catheters. Am J Med.

[197] Kim S-H, Kang C-I, Kim H-B et al (2003) Outcomes of Hickman catheter salvage in febrile neutropenic cancer patients with Staphylococcus aureus bacteremia. Infect Control Hosp Epidemiol. 10.1086/502157 10.1086/502157 14700404

[198] El Zakhem A, Chaftari AM, Bahu R (2014). Central line-associated bloodstream infections caused by Staphylococcus aureus in cancer patients: clinical outcome and management. Ann Med.

[199] Raad I, Hanna H, Boktour M (2004). Management of central venous catheters in patients with cancer and candidemia. Clin Infect Dis.

[200] Nucci M, Anaissie E (2001). Revisiting the source of candidemia: skin or gut?. Clin Infect Dis.

[201] Nucci M, Silveira MI, Spector N (1998). Risk factors for death among cancer patients with fungemia. Clin Infect Dis.

[202] Nucci M, Colombo AL, Silveira F et al (1998) Risk factors for death in patients with candidemia. Infect Control Hosp Epidemiol. 10.2307/30141563 10.1086/647743 9831941

[203] Nucci M, Anaissie E, Betts RF (2010). Early removal of central venous catheter in patients with candidemia does not improve outcome: analysis of 842 patients from 2 randomized clinical trials. Clin Infect Dis.

[204] Slavin MA, Sorrell TC, Marriott D (2010). Candidaemia in adult cancer patients: risks for fluconazole-resistant isolates and death. J Antimicrob Chemother.

[205] Liu CY, Huang LJ, Wang WS (2009). Candidemia in cancer patients: impact of early removal of non-tunneled central venous catheters on outcome. J Inf Secur.

[206] Janum S, Afshari A (2016) Central venous catheter (CVC) removal for patients of all ages with candidaemia. Cochrane Database Syst Rev 10.1002/14651858.CD011195.pub2 PMC6457908 27398809

[207] Ruhnke M, Cornely OA, Schmidt-Hieber M (2020). Treatment of invasive fungal diseases in cancer patients—revised 2019 recommendations of the Infectious Diseases Working Party (AGIHO) of the German Society of Hematology and Oncology (DGHO). Mycoses.

[208] Criscuolo M, Marchesi F, Candoni A (2019). Fungaemia in haematological malignancies: SEIFEM-2015 survey. Eur J Clin Investig.

[209] Lee YM, Moon C, Kim YJ, et al (2018) Clinical impact of delayed catheter removal for patients with central-venous-catheter-related Gram-negative bacteraemia. The Healthcare Infection Society 10.1016/j.jhin.2018.01.004 29330016

[210] Friedman ND, Korman TM, Fairley CK (2002). Bacteraemia due to Stenotrophomonas maltophilia: an analysis of 45 episodes. J Inf Secur.

[211] Boktour M, Hanna H, Ansari S (2006). Central venous catheter and Stenotrophomonas maltophilia bacteremia in cancer patients. Cancer.

[212] Hebeisen UP, Atkinson A, Marschall J, Buetti N (2019). Catheter-related bloodstream infections with coagulase-negative staphylococci: are antibiotics necessary if the catheter is removed?. Antimicrob Resist Infect Control.

[213] Raad I, Kassar R, Ghannam D (2009). Management of the catheter in documented catheter-related coagulase-negative staphylococcal bacteremia: remove or retain?. Clin Infect Dis.

[214] Wang CC, Mattson D, Wald A (2001). Corynebacterium jeikeium bacteremia in bone marrow transplant patients with Hickman catheters. Bone Marrow Transplant.

[215] Ghide S, Jiang Y, Hachem R (2010). Catheter-related Corynebacterium bacteremia: should the catheter be removed and vancomycin administered?. Eur J Clin Microbiol Infect Dis.

[216] Chaftari AM, El Zakhem A, Jamal MA (2014). The use of minocycline-rifampin coated central venous catheters for exchange of catheters in the setting of staphylococcus aureus central line associated bloodstream infections. BMC Infect Dis.

[217] Chaftari AM, Kassis C, El Issa H (2011). Novel approach using antimicrobial catheters to improve the management of central line-associated bloodstream infections in cancer patients. Cancer.

[218] Cesaro S, Cavaliere M, Spiller M (2007). A simplified method of antibiotic lock therapy for Broviac-Hickman catheters using a CLC 2000 connector device. Support Care Cancer.

[219] Funalleras G, Fernández-Hidalgo N, Borrego A (2011). Effectiveness of antibiotic-lock therapy for long-term catheter-related bacteremia due to gram-negative bacilli: a prospective observational study. Clin Infect Dis.

[220] Del Pozo JL, Cenoz MG, Hernáez S (2009). Effectiveness of teicoplanin versus vancomycin lock therapy in the treatment of port-related coagulase-negative staphylococci bacteraemia: a prospective case-series analysis. Int J Antimicrob Agents.

[221] Norris LAB, Kablaoui F, Brilhart MK, Bookstaver PB (2017). Systematic review of antimicrobial lock therapy for prevention of central-line-associated bloodstream infections in adult and pediatric cancer patients. Int J Antimicrob Agents.

[222] Gominet M, Compain F, Beloin C, Lebeaux D (2017). Central venous catheters and biofilms: where do we stand in 2017?. Apmis.

[223] Ahmad A, Moser C, Classen V (2019). Hydrochloric acid prolongs the lifetime of central venous catheters in haematologic patients with bacteraemia. Dan Med J.

[224] Wolf J, Connell TG, Allison KJ (2018). Treatment and secondary prophylaxis with ethanol lock therapy for central line-associated bloodstream infection in paediatric cancer: a randomised, double-blind, controlled trial. Lancet Infect Dis.

[225] Hachem R, Kanj S, Hamerschlak N (2018). International experience with minocycline, EDTA and ethanol lock for salvaging of central line associated bloodstream infections. Expert Rev Med Devices.

[226] O’Horo JC, Silva GLM, Safdar N (2011). Anti-infective locks for treatment of central line-associated bloodstream infection: a systematic review and meta-analysis. Am J Nephrol.

[227] Raad I, Chaftari AM, Zakhour R (2016). Successful salvage of central venous catheters in patients with catheter-related or central line-associated bloodstream infections by using a catheter lock solution consisting of minocycline, EDTA, and 25% ethanol. Antimicrob Agents Chemother.

[228] Beyar-Katz O, Dickstein Y, Borok S et al (2017) Empirical antibiotics targeting gram-positive bacteria for the treatment of febrile neutropenic patients with cancer. Cochrane Database Syst Rev 10.1002/14651858.CD003914.pub4 PMC6481386 28577308

[229] Raad II, Sabbagh MF (1992) Optimal duration of therapy for catheter-related Staphylococcus aureus bacteremia: a study of 55 cases and review. Clin Infect Dis. 10.1093/clinids/14.1.75 10.1093/clinids/14.1.75 1571466

[230] Pigrau C, Rodríguez D, Planes AM (2003). Management of catheter-related Staphylococcus aureus bacteremia: when may sonographic study be unnecessary?. Eur J Clin Microbiol Infect Dis.

[231] Stern A, Carrara E, Bitterman R et al (2019) Early discontinuation of antibiotics for febrile neutropenia versus continuation until neutropenia resolution in people with cancer. Cochrane Database Syst Rev 10.1002/14651858.CD012184.pub2 PMC6353178 30605229

